# On merging *Acer* sections *Rubra* and *Hyptiocarpa*: Molecular and morphological evidence

**DOI:** 10.3897/phytokeys.86.13532

**Published:** 2017-09-18

**Authors:** AJ Harris, Yousheng Chen, Richard T. Olsen, Sue Lutz, Jun Wen

**Affiliations:** 1 Smithsonian Institution, Department of Botany, MRC 166, Washington, D.C. 20013-7012 USA; 2 Institute of Botany, Chinese Academy of Sciences, Nanxincun 20, Xiangshan, Beijing 100093 China; 3 United States National Arboretum, 3501 New York Avenue, NE, Washington, D.C. 20002- 1958 USA

**Keywords:** *Acer laurinum*, *Acer pycnanthum*, *Acer rubrum*, *Acer saccharinum*, cuticle, ITS, scanning electron microscopy

## Abstract

In this study, we expanded Acer sect. Rubra Pax to include A. sect. Hyptiocarpa Fang. Traditionally, section Rubra comprises two iconic species, *Acer rubrum* Linnaeus (red maple) and *A. saccharinum* Linnaeus (silver maple), of eastern North American forests as well as the rare Japanese montane species, *A. pycnanthum* K. Koch. Section Hyptiocarpa consists of *A. laurinum* Hasskarl and *A. pinnatinervium* Merrill, which occur in subtropical and tropical regions of southwestern China to southeast Asia. Here, we confirm prior phylogenetic results showing the close relationship between sects. *Rubra* and *Hyptiocarpa*, and we use scanning electron microscopy to demonstrate that leaves of species within these sections have similar arrangements of cuticular waxes, which account for the silvery color of their abaxial surfaces. We describe that the sections also share labile sex expression; inflorescences that range from compound racemose thyrses, to racemes or umbels and that may have undergone evolutionary reduction; and several features of their fruits, such as seed locules without keels, basal portion of wings straight, acute attachment angle between mericarps, and production of some mericarps that are seedless and partially developed at maturity. Our expansion of sect. Rubra to include sect. Hyptiocarpa better elucidates the biogeographic and evolutionary history of these species. Additionally, we show that *A. laurinum* and *A. pinnatinervium* have intergrading morphology and are probably synonymous, but we note that further studies are required to conclude their taxonomic status.

## Introduction

*Acer* Linnaeus, the maple genus, is remarkable for comprising 125+ species and for representing one of the largest woody plant genera in the Northern Hemisphere next to oaks and willows (Ogata 1967; de Jong 1976; Murray 1970; Delendick 1981; Xu et al. 2008; van Gelderen et al. 1994; Weakley 2011). *Acer* exhibits a classical pattern of biogeographic disjunction across Europe, northern Africa, Asia, and North America with the greatest species richness in eastern Asia (Raven 1972; Thorne 1972; de Jong 1976; van Gelderen et al. 1994; Wen 1999; 2001; Qian and Ricklefs 2000; Xu et al. 2008; Wen et al. 2016; Harris et al. 2017). The genus is highly valued horticulturally and for timber and sugar products (Larsson and Jaciw 1967; Delendick 1981; van Gelderen et al. 1994; de Beaulieu and Mechelynck 2003; Barrett 2004).

*Acer* and the closely-related genus, *Dipteronia* Oliver (2 spp.), formerly comprised Aceraceae but are now treated in tribe Acereae of Sapindaceae (Acevedo-Rodriguez et al. 2011). Acereae belongs to subfamily Hippocastanoideae with tribe Hippocastaneae, which includes *Aesculus* Linnaeus, or the horsechestnuts and buckeyes, and two other small genera (Judd et al. 1994; Harrington et al. 2005; Buerki et al. 2010). Acereae has been fairly taxonomically stable, and consists of a well-supported clade based on morphological and molecular data (Willis 1980; Harrington et al. 2005; Pan et al. 2008; Buerki et al. 2010).

Within *Acer*, the circumscription of infra-generic groups has been controversial. Some groups are reasonably well agreed upon, such as section Macrantha, which includes species that have conspicuously white- or green-striped bark, and the recognition of *Acer carpinifolium* Sielbold & Zuccarini as the sole member of sect. Indivisia (e.g. Momotani 1962; Ogata 1967; Murray 1970; de Jong 1976; Delendick 1981; Wolfe and Tanai 1987; van Gelderen et al. 1994). On the other hand, some sections have been wildly unstable, such as sect. Negundo, which possesses one to several species and is sometimes raised to generic or subgeneric status (Fang 1966; de Jong 1976). The controversy regarding infra-generic groups in *Acer* ultimately reflects uncertainty about the evolutionary relationships among species.

Another maple that has not enjoyed taxonomic stability is *A. laurinum* Hasskarl. *Acer laurinum* was described as *A. javanium* (Junghuhn, 1841), an impressive tree with leaves and fruits that stood out from a distance. Hasskarl (1843) referred to Junghuhn’s description and renamed the taxon to *A. laurinum* (*nomen novum*) two years later, because the prior name was already in use (i.e., *A. javanicus*, now recognized as a species in either *Actinomorpha* or *Colona*, see Hasskarl 1857, Murray 1970). *Acer laurinum* has undergone considerable taxonomic splitting and lumping, and disagreements on its delimitation and the number of subspecific entities suggest that it is highly variable, but its variants may lack notable limits (e.g., Fang 1966; Murray 1970; van Gelderen et al. 1994). *Acer laurinum* is most often treated within sect. Hyptiocarpa (Fang 1966; = sect. Laurinum of Ogata 1967) or within sect. Integrifolia (Merrill 1941; Pax 1885). In the most recent global treatment of *Acer* van Gelderen et al. (1994) asserted that sect. Hyptiocarpa comprised *A. laurinum* and *A. garrettii* Craib, the latter of which the authors speculated may not merit species status. In contrast, Flora of China (Xu et al. 2008) represents a more recent and regional treatment of sect. Hyptiocarpa and recognizes two species: *A. laurinum* (including *A. garrettii*) and *A. pinnatinervium* Merrill, which the global treatment had synonymized with *A. garrettii*.

The affinities of sect. Hyptiocarpa remain highly speculative, and its closest relatives may be within sects. *Integrifolia*, *Trifoliata*, *Rubra*, or *Lithocarpa* (Pax 1885; Tanai 1978; Fang 1966; van Gelderen et al. 1994). *Acer laurinum* is an anomaly among maples and is of biogeographic importance, because it has a geographic range from southern China, Vietnam, and Thailand to the Philippines and Java. Therefore, it is the only maple to have a distribution that crosses the equator into the Southern Hemisphere.

**Figure 1.**
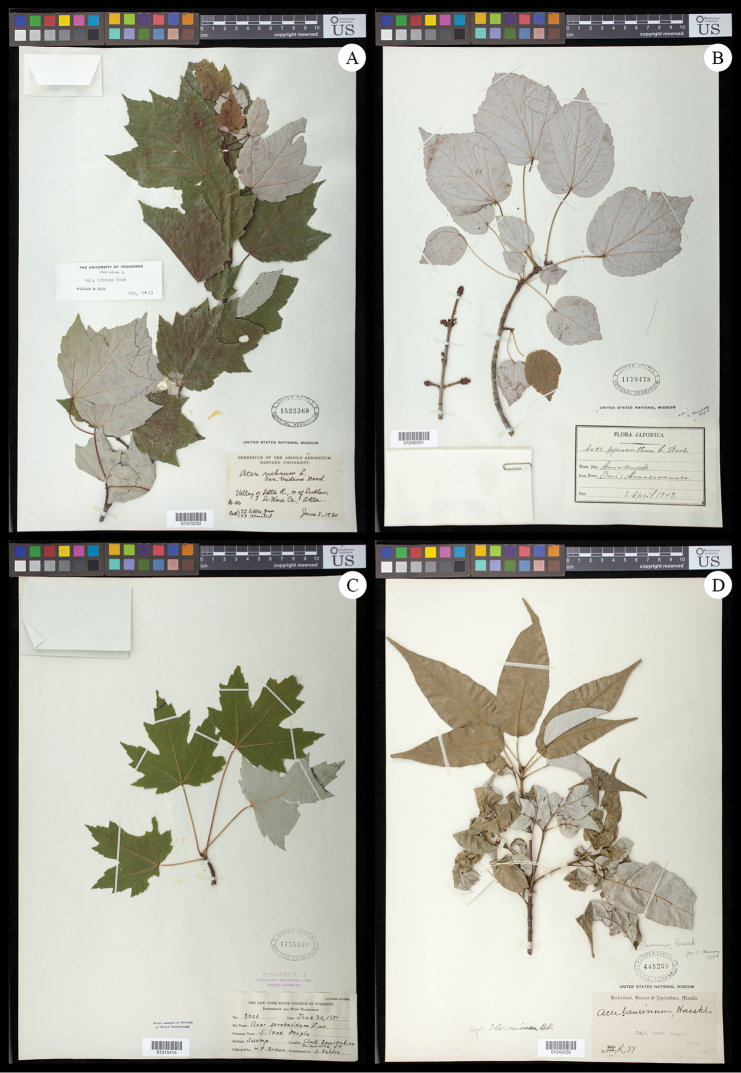
Typical specimens of *Acer* sects. *Rubra* and *Hyptiocarpa*, especially exhibiting leaf macromorphology. **A** *A. rubrum* **B** *A. pycnanthum* **C** *A. saccharinum* **D** *A. laurinum*. Specimens deposited at US national herbarium, and accession information visible in images. Detailed specimen records are available via the US online catalog (http://collections.nmnh.si.edu/search/botany/).

Recently, phylogenetic studies using chloroplast and nuclear DNA with several methods of analysis have repeatedly shown strong support for the somewhat unexpected sister relationship between *Acer* sects. *Hyptiocarpa* and *Rubra* (Suh et al. 2000; Tian et al. 2002; Grimm et al. 2006; Renner et al. 2008). Section Rubra is a highly supported clade (Grimm et al. 2006; Renner et al. 2008) that possesses three species: *A. rubrum* Linnaeus (red maple) and *A. saccharinum* Linnaeus (silver maple), which are iconic species in eastern North America, and *A. pycnanthum* K. Koch, which occurs in montane areas of Honshu in Japan (Ohwi 1965; van Gelderen et al. 1994). The molecular phylogenetic studies that support the relationship between sects. *Hyptiocarpa* and *Rubra* have included sequences from all species of sect. Rubra and from *A. laurinum* or, in one case, a sequence from a specimen of *A. garrettii* collected by its authority, Craib (Suh et al. 2000; Tian et al. 2002; Grimm et al. 2006; Renner et al. 2008). Infrequently, taxonomic and morphological studies have also speculated on a possible relationship between sects. *Rubra* and *Hyptiocarpa* (Delendick 1981; van Gelderen et al. 1994), and we observed that the possibility is reinforced by the conspicuous silvery abaxial leaf surfaces that are common to all four species (Fig. 1) and noted as a distinctive feature of sect. Hyptiocarpa (Merrill 1941).

In this study, we present evidence for the relationship between sects. *Rubra* and *Hyptiocarpa* from nuclear and chloroplast phylogenies and from an analysis of leaf cuticular wax ultrastructures. We also compare other morphological features of the sections according the available literature and specimens and discuss these in the context of biogeography and evolutionary radiation. Based on the results of our study, we propose combining sects. *Rubra* and Hyptiocarpa within sect. Rubra s.l. Throughout the study, we apply the taxonomy of van Gelderen et al. (1994) except as otherwise noted and for sect. Hyptiocarpa, for which we apply the more recent treatment from the Flora of China (Xu et al. 2008).

## Methods

### Phylogenetic reconstruction

We reconstructed phylogenies of Acereae at the section-level using sequences of nuclear Internal Transcribed Spacer (ITS) and the chloroplast spacer *trnD-psbM* (hereafter, *psbM*). We selected these markers because of their utility as DNA barcodes in plants (Dong et al. 2012; Li et al. 2011; Shaw et al. 2005; Zuo et al. 2011, 2017), their demonstrated utility in *Acer* for the sections in question (Grimm et al. 2006; Renner et al. 2008), and our preliminary observations about the information content of *psbM* for Acereae. We reconstructed phylogenies for this study even though prior studies have generated phylogenies of Acereae using chloroplast DNA, ITS, and nuclear genes (Suh et al. 2000; Tian et al. 2002; Grimm et al. 2006; Buerki et al. 2010; Renner et al. 2008; Harris et al. 2017), because doing so gave us more control over sampling of representative species, the ability to curate data and make decisions about data quality, and firsthand knowledge of all analysis parameters.

We obtained sequences of *psbM* and ITS from GenBank for representative samples of sections of *Acer* *sensu* Wolfe and Tanai (1987), *A. laurinum*, and all species of sect. Rubra *sensu* van Gelderen et al. (1994). We used Wolfe and Tanai (1987) to guide our taxonomic sampling, because their treatment splits *Acer* into smaller sections, which are more consistent with large, published molecular phylogenies (e.g., Grimm et al. 2006, Renner et al. 2008) than the most recent treatment by van Gelderen (1994). Wolfe and Tanai recognized 21 sections of extant *Acer*, and maintained *A. saccharinum* in a separate, monotypic section from *A. rubrum* and *A. pycnanthum*. Our section-level sampling according to Wolfe and Tanai (1987) may underrepresent diversity in *Acer*, especially within sect. Acer, which has been the least taxonomically stable section and probably includes species that are phylogenetically distant (Ogata 1967; Wolfe and Tanai 1987; van Gelderen et al. 1994; Grimm et al. 2006, Renner et al. 2008). Nevertheless, resolving relationships in sect. Acer is beyond the scope of our study and, based on outcomes from prior molecular phylogenetic studies (Grimm et al. 2006, Renner et al. 2008), species variously treated in sect. Acer are distant from sects. *Rubra* and *Hyptiocarpa*. Of the 21 sections recognized by Wolfe and Tanai, we sampled 20, but the missing section, sect. Integrifolia, may be represented by *Acer pentaphyllum* Diels. Wolfe and Tanai (1987) treated *A. pentaphyllum* in sect. Acer, but the species is included in sect. Pentaphylla in van Gelderen et. al (1994) with other species of Wolfe and Tanai’s (1987) sect. Integrifolia and is resolved with species of sect. Integrifolia in molecular phylogenies (Suh et al. 2000; Grimm et al. 2006; Renner et al. 2008). For all sections of *Acer*, we sampled the type species when possible. In addition to species of *Acer*, we included both species of *Dipteronia* in our analyses, and we utilized one sequence each of *Sapindus* Linnaeus, *Koelreuteria* Laxmann, and *Xanthoceras sorbifolium* Bunge as outgroups. *Xanthoceras* may be sister to all Sapindaceae and *Sapindus* and *Keolreuteria* represent the core Sapindaceae (*sensu* Buerki et al. 2010), which is sister to Hippocastanoideae (Buerki et al. 2010). We did not include Hippocastaneae among the outgroup or ingroup, because it has ITS sequences that are very difficult to align with *Acer* according to a prior report (Grimm et al. 2006) and our personal experience. Nevertheless, prior molecular phylogenetic studies of *Acer* have used cpDNA and have included *Aesculus* of Hippocastaneae (Renner et al. 2008; Tian et al. 2002). Therefore, we compare results of those studies with our own. The ITS and *psbM* datasets comprised 27 sequences each. The details of our sampling, including additional explanation of taxonomic representativeness and GenBank accession numbers, are presented in Table 1.

**Table 1.** Representative sampling of species used in this study for molecular phylogenetic analysis.

	Species	GenBank- ITS	GenBank- *psbM*	Section affiliation *sensu* van Geldren (1994)	Section affiliation *sensu* Wolfe and Tanai (1987)
Acereae	*Acer argutum* Maximowicz	AF401153	DQ659842	*Glabra*	*Arguta*
	*Acer campestre* Linnaeus	LK022558	DQ659844	*Platanoidea*	*Campestria*
	*Acer carpinifolium* Siebold & Zuccarini	AF401148	DQ659845	*Indivisia*	*Indivisa*
	*Acer cissifolium* (Siebold & Zuccarini) K. Koch	AY605402	KY682748	*Negundo*	*Cissifolia*
	*Acer distylum* Siebold & Zuccarini	DQ238354	DQ659850	*Parviflora*	*Distyla*
	*Acer glabrum* Torrey	DQ23834	DQ659892	*Glabra*	*Glabra*
	*Acer laurinum* Hasskarl	AF241490	DQ659854	*Hyptiocarpa*	*Laurina*
	*Acer macrophyllum* Pursh	DQ238352	DQ659860	*Lithocarpa*	*Macrophylla*
	*Acer negundo* Linnaeus	AY605407	DQ659864	*Negundo*	*Negundo*
	*Acer nipponicum* H. Hara	DQ366143	DQ659865	*Parviflora*	*Parviflora*
	*Acer palmatum* Thunberg	KT160159	DQ659867	*Palmata*	*Palmata*
	*Acer pensylvanicum* Linnaeus	AY605398	DQ659869	*Macrantha*	*Macrantha*
	*Acer pentaphyllum* Diels	DQ238478	DQ659870	*Pentaphylla*	*Acer*
	*Acer pentapomicum* Stewart ex Brandis	-	DQ659888	*Pubscentia*	*Pubscentia*
	*Acer pilosum* Maximowicz	DQ238345	-	*Pubscentia*	*Pubscentia*
	*Acer platanoides* Linnaeus	LK022676	DQ659871	*Platanoidea*	*Platanoidea*
	*Acer pseudoplatanus* Linnaeus	AM238269	DQ659872	*Acer*	*Acer*
	*Acer pycnanthum* K. Koch	AM113529	DQ659873	*Rubra*	*Rubra*
	*Acer rubrum* Linnaeus	AJ634580	DQ659874	*Rubra*	*Rubra*
	*Acer saccharinum* Linnaeus	AM113537	DQ659875	*Rubra*	*Eriocarpa*
	*Acer spicatum* Lamarck	AJ634578	DQ659879	*Parviflora*	*Spicata*
	Acer sterculiaceum K. Koch subsp. franchettii (Pax) A.E. Murray	DQ366145	KY682749	*Lithocarpa*	*Lithocarpa*
	Acer tataricum L. subsp. ginnala (Maximowicz) Maximowicz	AY605364	DQ659855	*Ginnala*	*Trilobata*
	*Dipteronia dyeriana* Henry	AM182900	DQ659838	-	-
	*Dipteronia sinensis* Oliver	AY605292	DQ659839	-	-
Outgroups	*Koelreuteria* Laxmann	EU72057	DQ659835	-	-
	*Sapindus* Linnaeus	AY207570	DQ659836	-	-
	*Xanthoceras* Bunge	FJ375202	DQ659837	-	-

*Notes *: We indicate the section affiliation of the species according to van Gelderen et al. (1994) and Wolfe and Tanai (1987), who largely followed Ogata (1967). We include the GenBank accession number for the sequences of ITS that we used for phylogenetic analyses. All species typify the sections recognized by Wolfe and Tanai (1987) except as follows: (A) We included *Acer pycnanthum* of sect. *Rubra* even though it does not typify the section, because of the objectives of the study; (B) We included *A. pentaphyllum* of sect. *Pentaphyllumsensu* van Gelderen et al. to stand in as a representative of sect. *Integrifolia* of Wolfe and Tanai. *Acer pentaphyllum* typifies sect. *Pentaphyllum* in van Gelderen et al. and Ogata, and van Gelderen’s sect. *Pentaphyllum* also includes species of Ogata’s sect. *Integrifolia*. While Wolfe and Tanai recognized sect. *Integrifolia*, they did not recognize sect. *Pentaphyllum*, and they treated *A. pentaphyllum* in sect. *Acer*; (C) In the *psbM* dataset, we utilized *A. garrettii* to stand in for *A. laurinum*, because there were no available sequences of *psbM*. *Acer garrettii* was not mentioned (and probably not recognized) by Ogata and was hesitantly given species status by van Gelderen.

Two sequences were new to this study: *psbM* of Acer sterculiaceum K. Koch subsp. franchettii (Pax) A.E. Murray and *A. cissifolium* (Siebold & Zucc.) K. Koch. We obtained the new sequences using fresh material, which we collected from the United States National Arboretum. Our collections consisted of leaves for DNA extractions, which we preserved in silica at the time of sampling, and voucher specimens, which we deposited at the United States National Herbarium (US; http://n2t.net/ark:/65665/396759747-a431-4859-b4a7-8c57db1cc2a2 and http://n2t.net/ark:/65665/36583930c-3354-4039-9e29-f9e0f9699ecb). We performed DNA extractions using a Qiagen Plant Mini Kit (Venlo, Netherlands) according to manufacturer recommendations, and we amplified *psbM* using forward and reverse PCR primers from Lee and Wen (2004). We performed PCR, sequencing, and purification steps using the reactions, thermocycling scheme, and protocols reported in Harris et al. (2017), except that the thermocycling included 35, rather than 40, cycles. Our primers for sequencing were the same as those that we used for PCR amplification. We reported the new sequences to GenBank (Table 1).

We performed sequence alignment using the MAFFT algorithm (Katoh et al. 2002; Katoh et al. 2005) on the GUIDANCE 2 (Sela et al. 2015) webserver (http://guidance.tau.ac.il/ver2/; Penn et al. 2010). GUIDANCE 2 helps to identify uncertain regions of an alignment by comparing alignments derived from bootstrap guide trees. The GUIDANCE 2 webserver also facilitates removing uncertain portions of an alignment and realigning through an iterative, interactive process. We performed initial alignments on our ITS and *psbM* data matrices with up to five MAFFT iterations for refinement and 100 bootstrap replicates. We used a conservative confidence score of 0.853 (GUIDANCE 2 Overview, http://guidance.tau.ac.il/ver2/overview.php), and we removed all sites with lower confidence scores. Following this step, we performed a new alignment in GUIDANCE 2 with the uncertain sites excluded, and we checked that the new alignment had a confidence score of at least 0.95 (out of 1.0 possible) averaged across all sites. We also checked the final alignment visually with sites color-coded according to their GUIDANCE 2 score using JALVIEW (Waterhouse et al. 2009) on the GUIDANCE 2 webserver. We concatenated aligned matrices using SEQUENCEMATRIX (Vaidya et al. 2011), and our concatenated matrix comprised composite taxonomic entities of the same section in *Acer* and usually of the same species, except in the case of sects. *Rubra* and *Hyptiocarpa*, for which composite entities were always of the same species (see Table 1 for *Dipteronia* and outgroups). We provide all final alignments in Dryad: http://dx.doi.org/10.5061/dryad.n26nd

Prior to phylogenetic analyses, we assessed the data matrices for base compositional heterogeneity and to determine the best nucleotide substitution model. We sought to detect base compositional heterogeneity, because it can lead to errors in phylogenetic inferences especially in the placement of outgroups and other long branches (Tarrío et al. 2000; Jermiin et al. 2004; Sheffield 2013). We performed the analysis for base compositional heterogeneity using a chi square test in PAUP* (Swofford 2002). We estimated the best model of nucleotide substitution from among 1-, 2-, and 6- parameter models with and without gamma rate variation (see Yang 1996 regarding invariance) in JMODELTEST (Posada 2008) under the Bayesian information criterion (BIC), and determined that the 6-parameter SMY+G (BIC=6544.4) and 2-parameter K80+G (BIC=5387.7) models were the best fit for ITS and *psbM*, respectively.

We performed phylogenetic analyses using neighbor-joining (NJ), maximum likelihood (ML), and Bayesian inference (BI) methods independently for ITS and *psbM* as well as for the concatenated data matrix. We performed the NJ analyses in GENEIOUS TREE BUILDER using Jukes Cantor distance and 1000 NJ bootstrap (BS) replicates to assess support. We reconstructed the ML trees in MEGA 6.06 (Tamura et al. 2013). In MEGA, we set models according to the results from JMODELTEST except that we used GTR+G for ITS, because it is the only 6-parameter model available in MEGA. We performed the analyses with five gamma rate categories and the subtree pruning and recrafting method of branch swapping. We also performed 500 BS replicates under the same parameters to determine support for clades. For BI, we utilized the GTR+G model of nucleotide substitution *a priori* (see Huelsenbeck and Rannala 2004; Ronquist et al. 2011) and unlinked models for the two markers in the analysis of concatenated data. The BI analysis comprised two simultaneous runs of 20 million generations with 12 incrementally heated MCMC chains each in MRBAYES 3.2.6 (Ronquist and Huelsenbeck 2003; Ronquist et al. 2011; Ronquist et al. 2012). We sampled the MCMC every 5000 generations and used Tracer 1.6 (Rambaut and Drummond 2007) to confirm stationarity and that a 10% burnin per independent analysis was appropriate. We combined results for simultaneous analyses using LOG COMBINER of the BEAST 1.8.0 software package (Drummond and Rambaut 2007; Drummond et al. 2012). We summarized the combined trees for each gene by selecting a maximum clade credibility tree with TREE ANNOTATOR, also of the BEAST 1.8.0 software package, and we obtained branch lengths for the selected tree using the median lengths from among the posterior distribution of trees. We also generated alternative summaries of the combined BI trees in GENEIOUS using 50% majority rule consensus with compatible groups with less than 50% support allowed. We visualized and rooted the final NJ, ML, and summarized BI trees in FIGTREE 1.4.0 (Rambaut and Drummond 2009). All final trees with clade support values are available in Dryad: http://dx.doi.org/10.5061/dryad.n26nd.

### Examination of cuticular wax ultrastructure of leaves

For the morphological study of leaves, we examined individuals representing all four species comprising *Acer* sects. *Rubra* and *Hyptiocarpa*. We sampled leaves from all available specimens of *A. laurinum* and *A. pycnanthum* and four specimens each of *Acer rubrum* and *A. saccharinum* (Table 2). Our sampling of *A. rubrum* and *A. saccharinum* included mid- and late-season specimens from two or more geographically distant parts of the ranges of the species and was designed to facilitate detection of population-level and seasonal variation in cuticular wax features (Sargent 1922; de Jong 1976; Delendick 1981). We obtained leaf samples near the center of leaves from sites adjacent to the midvein. The samples were dry when we obtained them from herbarium sheets. Air-dried samples, such as from herbarium sheets, are suitable for examination of cuticles without additional preparations and do not typically develop structural artifacts from drying or during examination with SEM (Pathan et al. 2010). We used specimens deposited at the United States National Herbarium (US) to obtain all leaf materials (Table 2).

**Table 2.** Specimens of *Acer* sections *Rubra* and *Hyptiocarpa* from which we obtained leaf material for study. All specimens are deposited at the United States National Herbarium (US; http://collections.nmnh.si.edu/search/botany/). Locations are given as state/province, county or with as much information as is available. Refer to Table 1 for taxon authorities.

Species	Collector name and number	Location	Stable URL to online specimen record
*Acer laurinum*	*Cult., in Hort. Bog. III,K 37*	Island of Java, Indonesia	http://n2t.net/ark:/65665/36a9749fe-e79a-456c-86bd-cb7d9ff2f6d2
*Acer laurinum*	*Sandkuhl 21296*	Benguet, Philippines	http://n2t.net/ark:/65665/3537d77b8-a9eb-460d-bd60-e16ce673f833
*Acer laurinum*	*Wen 13386*	Guangzhou (Cult.), China	http://n2t.net/ark:/65665/3b7b44164-fff4-4d7f-99a4-5d3d7adc2cbc
*Acer pycnanthum*	*Wilson 7729*	Island of Honshu, Japan	http://n2t.net/ark:/65665/369db8fdf-3e39-4f54-91d9-d8b631a4455f
*Acer pycnanthum*	*Wilson 6882*	Island of Honshu, Japan	http://n2t.net/ark:/65665/3744ee9ab-cddd-4013-9b52-cbda00eb02e3
*Acer pycnanthum*	unknown	Omi, Japan	http://n2t.net/ark:/65665/3255f723b-0c91-495a-94cc-c4d058c90352
*Acer rubrum*	*Arsene 11583*	Louisiana, USA	http://n2t.net/ark:/65665/37fa4f77a-b5eb-4c61-95ec-d967147cc603
*Acer rubrum*	*Thieret 22942*	Louisiana, USA	http://n2t.net/ark:/65665/3b286464c-77be-4e11-bf53-aaf52e3f8299
*Acer rubrum*	*Stevens 2617*	Oklahoma, USA	http://n2t.net/ark:/65665/3c46d528c-6d03-4ab4-abfc-34c8b6821ceb
*Acer rubrum*	*Little & Ulmsted 186*	Oklahoma, USA	http://n2t.net/ark:/65665/39b4b7e0b-6ebc-46f9-b95a-d46b60d635ec
*Acer rubrum*	*Harris 2016-63*	West Virginia, USA	http://n2t.net/ark:/65665/329687fb5-8d3b-4d04-906e-c85a93d58334
*Acer saccharinum*	*Brown 8023*	New York, USA	http://n2t.net/ark:/65665/37328cb10-f106-4a89-a265-b7d8ade2aff7
*Acer saccharinum*	*Coville s.n.*	New York, USA	http://n2t.net/ark:/65665/3e52409ce-5840-4236-ad62-51434f8259a7
*Acer saccharinum*	*Richardson & Robertson 915*	Kansas, USA	http://n2t.net/ark:/65665/302ee09b6-3742-4616-b6fe-1af6c08c1e12
*Acer saccharinum*	*Norton 69*	Kansas, USA	http://n2t.net/ark:/65665/3f46187ca-30f8-442b-a449-c90d7a2f6807

We used a Hitachi TM300 scanning electron microscope (SEM) to examine the ultrastructure of the abaxial and adaxial surfaces of the leaves following standard protocols. We used a standard working depth of 10mm and took SEM micrographs under 15kv after determining that this intensity of the electron beam would not melt the cuticular wax. All of our scanning electron micrographs of the leaf surfaces are available from in Dryad: http://dx.doi.org/10.5061/dryad.n26nd.

Throughout, we apply the term ‘cuticle’ to all parts of the wax layer(s) above the cellulose wall of the epidermal cells. We acknowledge that the cuticle is a complex structure comprised of many well-delimited and/or intergrading components (reviewed in Fernández et al. 2016). However, our imaging is from a birdseye view, such that we are not able to distinguish among cuticular layers. We use terminology for cuticular wax forms following Barthlott et al. (1998). For discussion of leaf characters, especially veins, we follow The Manual of Leaf Architecture of the Leaf Architecture Working Group (1999).

### Examination of specimens

We examined numerous herbarium specimens to complete this study. In particular, we examined specimens in person at US, South China Botanical Garden (IBSC), and the United States National Arboretum (NA). We also examined high resolution images of specimens online using JSTOR Global Plants (http://plants.jstor.org/) and SEINet (http://swbiodiversity.org/seinet).

## Results

The aligned sequence matrices of ITS and *psbM* (http://dx.doi.org/10.5061/dryad.n26nd) each had alignment scores of 0.96. The ITS matrix comprised 564 characters, and *psbM* had 856 characters. Neither *psbM* nor ITS had significant differences in base composition (*χ2* crit = 10.2, *p*=1.0 and *χ2* crit = 23.8, *p*=1.0, respectively).

**Figure 2.**
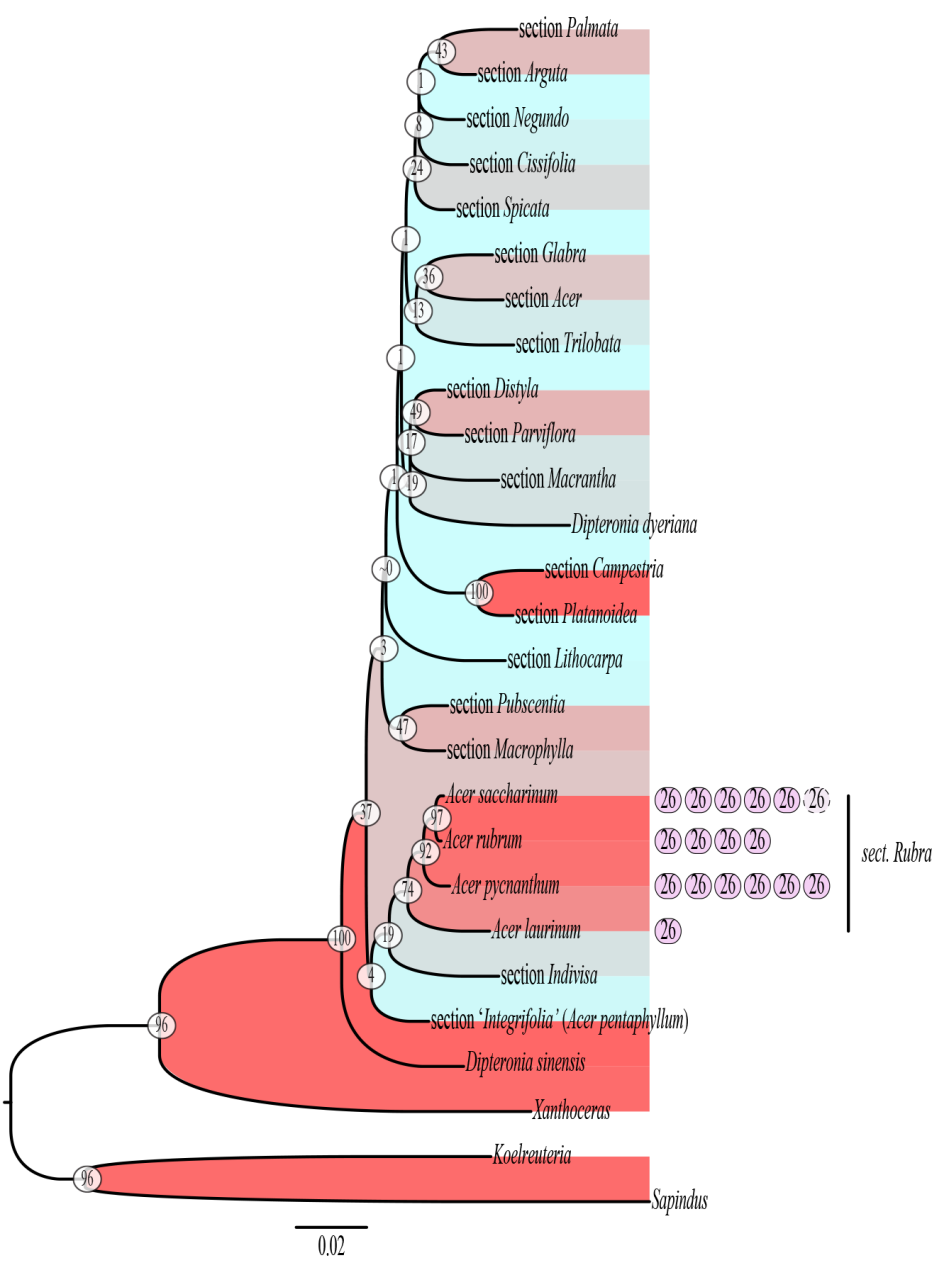
Maximum likelihood tree. Composite individuals represent sections except in the case of sects. *Rubra* and *Hyptiocarpa*, where composite individuals represent species. See Table 1 for GenBank accession numbers. Boostrap values for nodes are shown in white circles. Purple circles to the right of species in sects. *Rubra* and *Hyptiocarpa* represent one set each of 26 chromosomes (i.e., 2n=26) and show ploidy levels in sects. *Rubra* and *Hyptiocarpa* (e.g., *A. saccharinum* is tetraploid). Color coding of red and blue among branches shows relative support, respectively, from high (=100%BS) to low (~0%BS). Branches are scaled according to the bar below the tree.

Phylogenetic analyses of ITS showed weak support for the monophyly of the clade comprising sects. *Rubra* and *Hyptiocarpa*: NJ BS 45%, BI posterior probability (PP) 0.78, ML BS 48%. The *psbM* data matrix had few informative characters to distinguish a clade of sects. *Rubra* and *Hyptiocarpa* from *Acer distylum* Siebold & Zucc. of the monotypic sect. Distyla. Sections *Rubra* and *Hyptiocarpa* formed a trivially supported clade in the NJ phylogeny. However, a clade of sects. *Rubra* and *Hyptiocarpa* included *A. distylum* in the ML phylogeny. The BI results from *psbM* highlight the low support for the relationships among sects. *Rubra*, *Hyptiocarpa*, and *Distyla* in that the maximum clade credibility summary showed a clade of sects. *Rubra* and *Hyptiocarpa*, while the majority rule summary showed *A. distylum* included in a clade with sects. *Rubra* and *Hyptiocarpa*. The concatenated data matrix of ITS and *psbM* yielded moderate support for a clade of sects. *Rubra* and *Hyptiocarpa* in NJ, ML, and BI analyses. The support for the *Rubra*-*Hyptiocarpa* clade was NJ BS of 45%, BI PP of 0.74 in the maximum clade credibility tree and of 0.71 in the majority rule topology, and ML BS of 74% (Fig. 2). All trees in which sect. Rubra and *Hyptiocarpa* form a clade suggest that *A. pycnanthum* is sister to a clade of *A. rubrum* and *A. saccharinum* and that *A. laurinum* is sister to the other three species.

**Figure 3.**
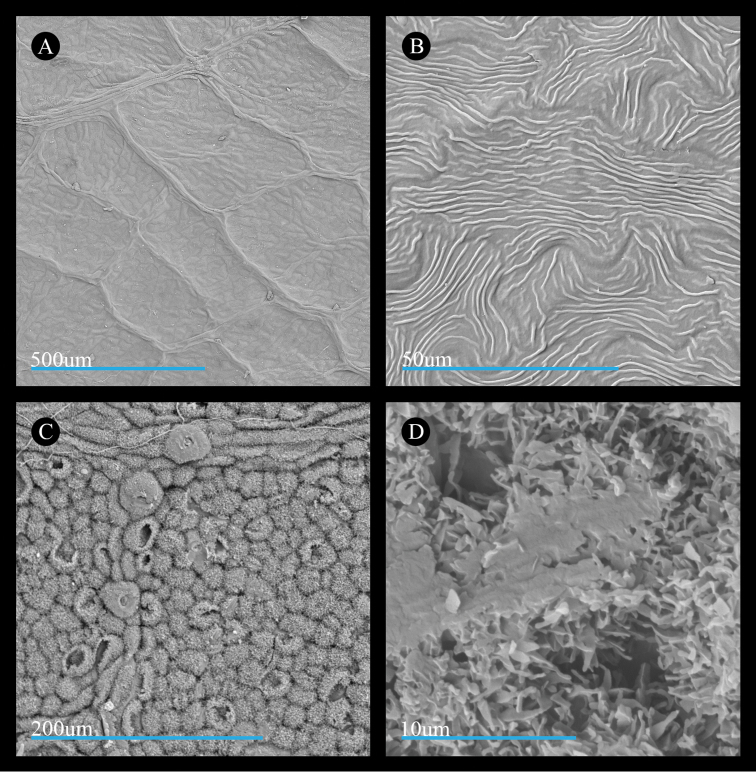
Micrographs of the leaf surfaces of *Acer rubrum*. **A** Adaxial surface showing cell shape and organization (*Thieret 22942*) **B** Adaxial surface showing cuticle (*Harris 2016-63*) **C**, Abaxial surface showing cell shape and organization of cells and stomata (*Stevens 2617*) **D** Abaxial surface showing cuticular wax (*Thieret 22942*). All leaf materials are from specimens deposited at US, and parenthetical information in this legend refers to the collector name and number for the source specimen.

Our examination of leaf surface features in *Acer rubrum* shows that the adaxial surface bears pavement cells that are generally ovoid in shape and have wavy, jig-saw puzzle-piece-like margins (Fig. 3A–B). Across the surfaces of the pavement cells, the cuticle comprises a smooth layer and forms a striate pattern (Fig. 3B). The abaxial surfaces of leaves in *Acer rubrum* bear a cuticular wax layer comprised of membranous platelets, for which the membranes sometimes coalesce into structures appearing as terraced or non-terraced wax splatters and finger-like extensions of the membranes are thin, polygonal (Fig. 3C–D).

**Figure 4.**
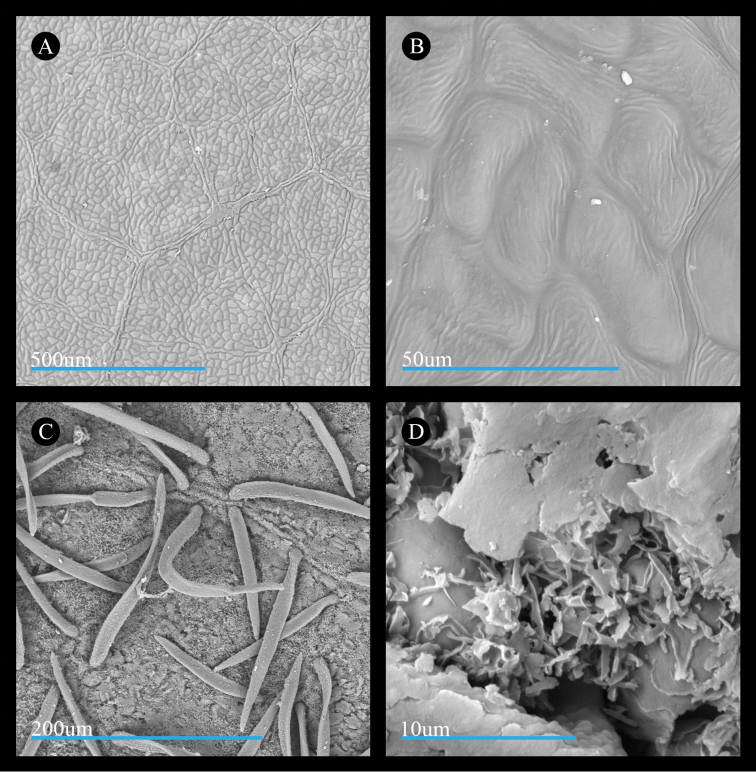
Micrographs of the leaf surfaces of *Acer saccharinum*. **A** Adaxial surface showing cell shape and organization (*Norton 69*) **B** Adaxial surface showing cuticle (*Richardson & Robertson* 915) **C** Abaxial surface showing cell shape and organization of cells and stomata (*Brown 8023*) **D** Abaxial surface showing cuticular wax (*Coville s.n.*). All leaf materials are from specimens deposited at US, and parenthetical information in this legend refers to the collector name and number.

Wax features of leaves of *Acer saccharinum* are similar to those of *A. rubrum*. Specifically, the adaxial surface bears wavy pavement cells (Fig. 4A–B) and the abaxial surface is covered by a cuticular wax layer comprised of membranous platelets with thin extensions (Fig. 4C–D). However, the wax splatter features formed by the coalescing of the membranous platelets appear larger and more frequent on the leaf surface (Fig. 4D).

**Figure 5.**
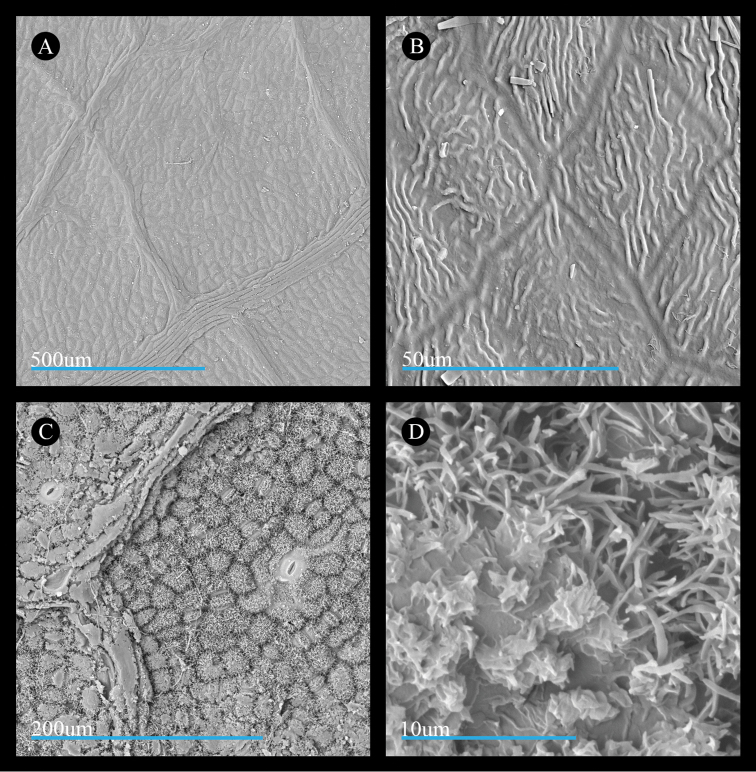
Micrographs of the leaf surfaces of *Acer pycnanthum*. **A** Adaxial surface showing cell shape and organization (*Wilson 6882*) **B** Adaxial surface showing cuticle (*Collector unknown, s.n.*) **C** Abaxial surface showing cell shape and organization of cells and stomata (*Wilson 7729*) **D** Abaxial surface showing cuticular wax (*Wilson 6882*). All leaf materials are from specimens deposited at US, and parenthetical information in this legend refers to the collector name and number..

Leaves of *Acer pycnanthum* bears wax similar to those of *A. rubrum* and *A. saccharinum* and show wavy pavement cells with striate cuticular wax (Fig. 5A–B). On its abaxial surface, *Acer pycnanthum* bears the coalescing membranous platelets (Fig. 5C–D). The platelets sometimes have rounded extensions instead of or alongside of polygonal ones (compare raw images provided in http://dx.doi.org/10.5061/dryad.n26nd).

**Figure 6.**
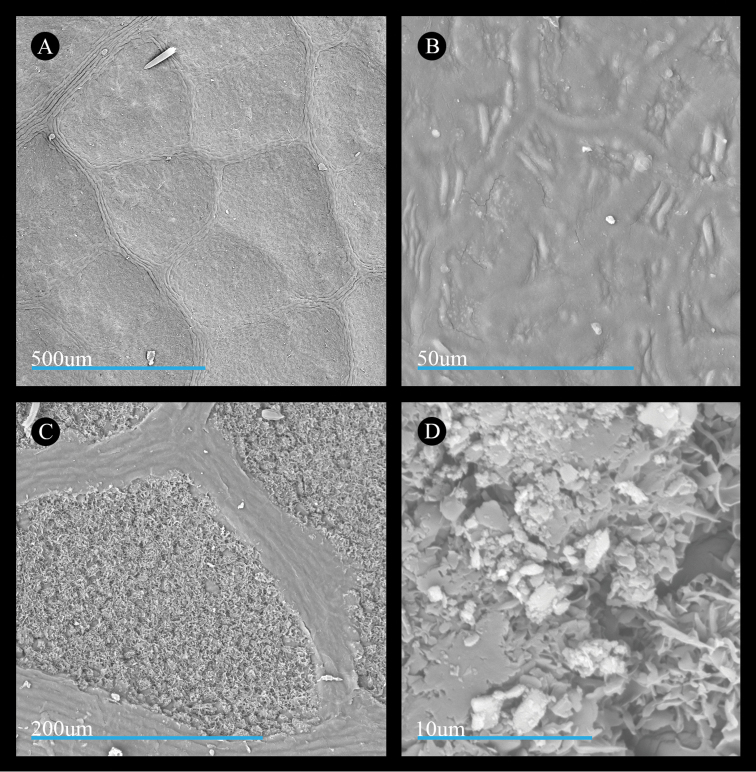
Micrographs of the leaf surfaces of *Acer laurinum*. **A** Adaxial surface showing cell shape and organization (*Cult., in Hort. Bog. III,K,37*) **B** Adaxial surface showing cuticle (*Sandkuhl 21296*) **C** Abaxial surface showing cell shape and organization of cells and stomata (*Sandkuhl 21296*) **D** Abaxial surface showing cuticular wax (*Wen 13386*). All leaf materials are from specimens deposited at US, parenetical information in this legend refers to the collector name and number.

In *Acer laurinum*, the cuticular smooth layer on the adaxial leaf surface has wrinkles that make it appear thicker than in species of sect. Rubra s.s. The smooth layer may be slightly or extensively wrinkled across the adaxial surface (Fig. 6A–B, also http://dx.doi.org/10.5061/dryad.n26nd), and it obscures the shapes of the pavement cells. The membranous platelets on the abaxial surface (Fig. 6C) are wide and do not taper into finger-like projections at their ends (Fig. 6D). The wax splatter feature is sometimes granular on its surface (Fig. 6D).

**Figure 7.**
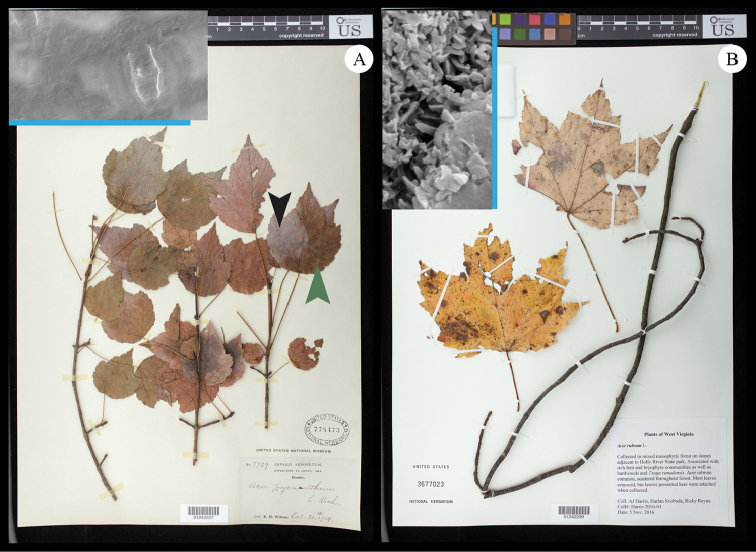
Late-season specimens of Acer sect. Rubrum approaching leaf senescence. **A** *A. pycnanthum* with black arrow indicating silvery abaxial surface and green arrow indicating non- silvery surface. The inset in the upper left shows an SEM micrograph of a portion of an abaxial leaf surface from this specimen that lacks the silvery color such as the area referred to by the green arrow. Blue scale bar = 50μm. For an SEM micrograph showing a silvery portion of leaf surface from this specimen, see Figure 5C **B** *A. rubrum* exhibiting exclusively silvery abaxial leaf surface (upper, right leaf). The inset in the upper left shows an SEM micrograph of a portion of an abaxial leaf surface from this specimen bearing the characteristic silvery surface, and we did not observe late season leaves of *A. rubrum* lacking the silvery surface. Herbarium specimens deposited at US, and accession information visible in images. Detailed specimen records are available via the US online catalog (http://collections.nmnh.si.edu/search/botany/).

We did not detect differences in the leaf wax features based on geographic range or, in most cases, seasonality. However, we observed one late-season *Acer pycnanthum* specimen with some leaves partially lacking the silvery color on the abaxial surface (Fig. 7A). The silvery portion showed cuticular waxes similar to those on the mid-season leaves (Fig. 5B, see also http://dx.doi.org/10.5061/dryad.n26nd) while the non-silvery portion clearly lacked the plate-like cuticular features altogether (Fig. 7A, inset). However, on an equivalently late-season specimen of *A. rubrum* (Fig. 7B), we did not observe any parts of the leaves lacking the silvery component and membranous plates appeared the same as on the mid-season leaves (Fig. 7B, and insert).

## Discussion

### Phylogenetic relationship between Acer sections *Rubra* and *Hyptiocarpa*

Our phylogenetic results are congruent with previous molecular studies, which have found well-supported close relationships between *Acer* sects. *Rubra* and *Hyptiocarpa*. For example, Renner et al. (2008) reconstructed a phylogeny of Acereae from six chloroplast genes, including *psbM*, and using all four species comprising sects. *Rubra* and *Hyptiocarpa*. They found 99%BS support for a *Rubra*-*Hyptiocarpa* clade based on an ML analysis and showed the same relationships within the clade as in our analyses (Fig. 2; data in Dryad) (Renner et al. 2008). Similarly, Li et al. (2006) performed an NJ analysis of Acereae and found 100%BS support for a clade of sects. *Rubra* and *Hyptiocarpa* according to two chloroplast genes, including *psbM*, and Grimm et al. (2006) obtained the same result using MP and BI analyses of ITS. Tian et al. (2002) also recovered the *Rubra*-*Hyptiocarpa* clade from concatenated ITS and one chloroplast gene, *trnL-F*, except that they did not include *Acer pycnanthum* in their study. In addition to phylogenetic reconstruction, network analyses have also shown strong support for the grouping of the *Rubra*-*Hyptiocarpa* (Grimm et al. 2006; Renner et al. 2008). By comparison to other studies, we found relatively low molecular phylogenetic support for the *Rubra*-*Hyptiocarpa* clade, and this is probably due to stringent removal of uncertain portions of our alignments and because our chloroplast dataset is small, comprising only one gene.

### Similarities in leaf wax features between *Acer* sections *Rubra* and *Hyptiocarpa*

We observed very similar cuticular wax configurations on the abaxial leaf surfaces of species of sect. Rubra and in *A. laurinum*. In general, these configurations comprised membranous crystals that coalesce in formations appearing as wax splatters on the surface. We unexpectedly showed evidence that cuticles comprised of membranous plates are the source of the classic silvery appearance in sects. *Rubra* and *Hyptiocarpa* by showing that when cuticular wax formation is absent in *A. pycnanthum* (Fig. 7A), so is the silvery color. We expect that cuticular waxes are probably responsible for the silvery color in all species of sects. *Rubra* and *Hyptiocarpa*, and the relationship between the silver color and cuticular waxes has been previously noted and explored (e.g., Baker 1974; Caddah et al. 2012).

Some authors have speculated that cuticular wax configurations may be of limited taxonomic value, because they could vary with environment (Baker 1974; Mayeux et al. 1981). However, cuticular waxes have been informative in other groups (e.g., *Jatropa* Linnaeus, Dehgan 1980; Rosa Linnaeus sect. Caninae, Wissemann 2000; and *Aralia* Linnaeus, Wen 2011) and often have clear evolutionary significance (Eglinton and Hamilton 1967). Moreover, we did not find notable differences in the waxes among specimens collected in different parts of their geographic ranges or during different seasons (compare images at http://dx.doi.org/10.5061/dryad.n26nd from specimens of *Acer rubrum* and *A. saccharinum*). The striking cuticular waxes on the abaxial surfaces of all four *Rubra*-*Hyptiocarpa* species probably reflects descent from a common ancestor and could function in insect interactions (e.g., limiting insect walking on the abaxial surfaces; Baker 1974; Eigenbrode and Espelie 1995; Federle et al. 1997; Gorb et al. 2008; Müller 2008) or reducing water loss (Sutter and Langhans 1982; Clarke and Richards 1988).

The cuticle layer on the adaxial surface of *Acer laurinum* appears less similar to the species of section Rubra. While both sects. *Rubra* and *A. laurinum* have striations, these differ in the size of the striae, or ridges, which are wider and taller in *A. laurinum* (compare Fig. 6B with Figs 3B, 4B, 5B). Additionally, the size of the striae in *A. laurinum* makes the cuticle appear thicker than in the other species. A thick cuticle in *A. laurinum* would be consistent with its distribution in subtropical and tropical regions (Bloembergen 1948; van Gelderen et al. 1994; Xu et al. 2008), as tropical species often exhibit thick cuticles to reduce leaching via regular rainfall (Martin and Juniper 1970; Boeger et al. 2004). Striations of different widths and heights between *A. laurinum* and sect. Rubra may represent specialized adaptations to local conditions but the presence of striations may arise from a common genetic architecture. Recent studies on the genetic basis for cuticular wax phenotypes in model organisms such as *Sorghum* L. (Punnuri et al. 2017) and *Arabidopsis* Heynh. (Lee and Suh 2015) provide a foundation for future investigations of the evolutionary origins of cuticular wax forms in sects. *Rubra* and *Hyptiocarpa* and other maples.

*Acer* sects. *Rubra* and *Hyptiocarpa* cannot be united strictly based on the appearance of the abaxial surfaces of their leaves. Although this feature may have taxonomic value (Merrill 1941; Krause 1978; Delendick 1981) and it appears monomorphic in sects. *Rubra* and *Hyptiocarpa*, it also occurs elsewhere in the genus (van Gelderen et al. 1994). In particular, silvery or glaucous surfaces occur in most species of sect. Pentaphyllum and in some species of sect. Acer. Nevertheless, the taxonomic informativeness of cuticular waxes in *Acer* may warrant further investigation to compare both the fine features of ultrastructure and wax chemical composition especially within and among glaucous and non-glaucous sections and species.

### *Acer laurinum* and other species of section Hyptiocarpa

Different taxonomic treatments of *Hyptiocarpa* do not all agree on species delimitation within the section. The large number of synonyms in *Hyptiocarpa* and confusion over the boundaries of species may reflect high variability and the need for additional field work to elucidate species limits or intergradation (Bloembergen 1948; van Gelderen et al. 1994). The most recent treatment of sect. Hyptiocarpa in Flora of China (Xu et al. 2008) recognizes two species: *Acer laurinum* and *A. pinnatinervium*. *Acer pinnatinervium* is considered a synonym of *A. laurinum* by van Gelderen (1994) and in the Plant List (http://www.theplantlist.org), but its status within *Hyptiocarpa* merits discussion here.

According to Xu et al. (2008), *Acer laurinum* and *A. pinnatinervium* differ in fruit size, the number of primary veins per leaf, and their geographic distributions. *Acer laurinum* has fruits 4-7 cm and leaves with three primary veins, while *Acer pinnatinervium* has fruits 2-4 cm and only one primary vein, i.e., it is truly pinnately veined. The pinnate venation in *Acer pinnatinervium* may be particularly noteworthy, because most species of *Acer* have leaves with three main veins (Merrill 1941). Therefore, pinnate venation in *A. pinnatinervium* is considered the primary character for distinguishing it from *A. laurinum* (Merrill 1941). With respect to geographic distributions, Xu et al. (2008) report that *Acer laurinum* has a broader range, being found from southwestern China to India, Vietnam, Indonesia, and the Philippines, while *A. pinnatinervium* occurs in southwest China, Thailand, and India.

Closer examination of *Acer laurinum* and *A. pinnatinervium* shows that they intergrade on the number of primary veins. Some collections of *A. laurinum* (e.g., *Blume 466*, L; *Blume s.n.*, L) show strong basal acrodromous veins, while isotypes of *Acer pinnatinervium* (*F. Kingdon-Ward 9102*, A, BM) show pinnate venation with brochidodromous secondary veins near the leaf base. However, the holotype and isotype of *A. laurinum* (*F.W. Junghuhn s.n.*, L, U, respectively) each show variability in venation such that some leaves have acrodromous veins and others are pinnately veined with weak brochidodromous secondaries. We also observed this variability within a specimen of *A. laurinum* utilized in the SEM component of this study, *Cult., in Hort. Bog. III,K,37* (see Table 2), and in many specimens that are ascribed to *A. pinnatinervium* and digitized in the Chinese Virtual Herbarium (http://www.cvh.ac.cn/). In the latter case, intra-individual variability of leaf veins may account for recent disagreements in the identities of specimens as either *A. laurinum* or *A. pinnatinervium* evidenced by the annotation labels. Based on these observations, we suspect that the number of primary veins is not be sufficient to distinguish *Acer pinnatinervium* from *A. laurinum*, and combining the two species may be needed pending an additional study of more strategically samples individuals.

### Morphology of *Acer* sections *Rubra* and *Hyptiocarpa*

**Figure 8.**
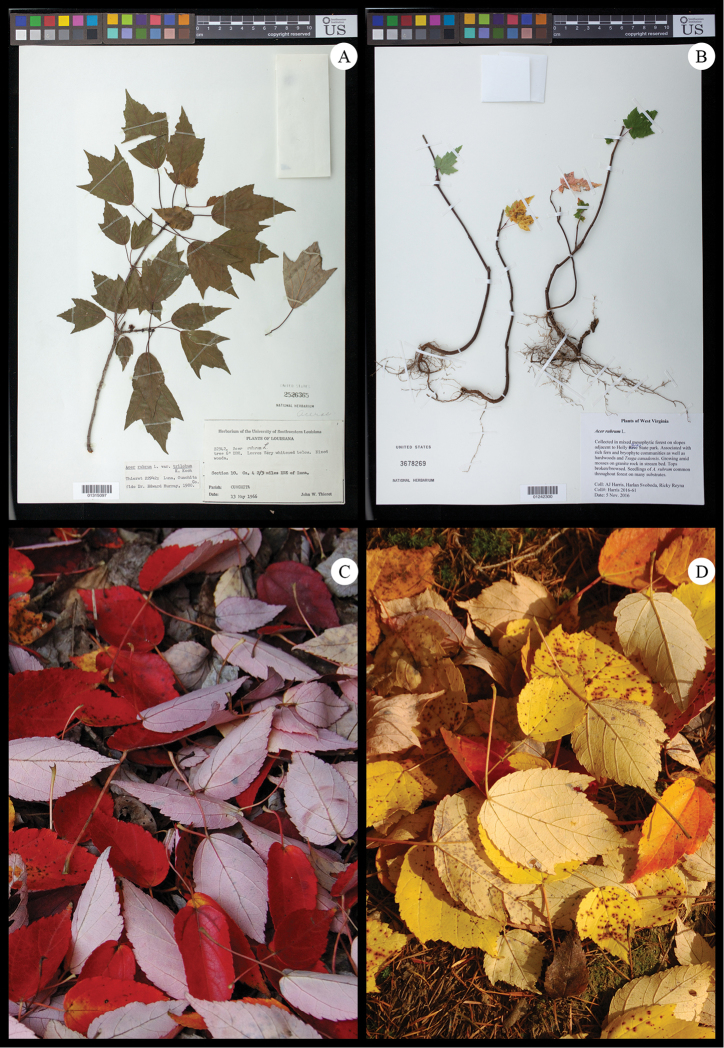
Elongate leaf shape in *Acer rubrum* and *A. pycnanthum*. **A–B** *A. rubrum* **C–D** *A. pycnanthum*. Unfortunately, there is no scale for the images of *A. pycnanthum*, but the leaf size is similar to that illustrated in Figure 1B. Herbarium specimens in **A** and **B** deposited at US, and accession information visible in images. Detailed specimen records are available via the US online catalog (http://collections.nmnh.si.edu/search/botany/).

Leaves in sects. *Rubra* and *Hyptiocarpa*, hereafter sect. *Rubra* *sensu latu*, exhibit shapes that vary within and among species from elongate to orbicular (Fig. 1). *Acer laurinium* and *A. pinnatinervium* have highly elongate leaves, while, in *A. rubrum*, leaves vary from being orbicular (Fig. 1A) to having slight elongation (Fig. 8A). Similarly, leaves in mature *A. pycnanthum* may also possess roughly orbicular leaves (Fig. 1B) to leaves that are highly elongated and nearly lacking lobes (Fig. 8C–D). In *A. saccharinum*, most individuals have leaves that are more-or-less orbicular (Fig. 1C), but some have elongated leaves (e.g., *Chaney 290*, LSU). Many species of *Acer* exhibit elongation of juvenile leaves, including in *A. saccharinum* (e.g., *Longbottom 8925*, DOV), *A. pycnanthum* (e.g., *Meyer 12513*, NA), and *A. rubrum* (Fig. 8B). Additionally, leaves and leaflets in other Sapindanceae are also often elongate (Acevedo-Rodríguez et al. 2011; Harris et al. 2017). Leaf elongation in seedlings of *Acer* may indicate an underlying genetic architecture in the genus and, consequently, ontogenic recapitulation (Haeckel 1866; e.g., Mishler 1998). Thus, while variable leaf shape in sect. Rubra does not unite its species, the tendency towards elongation is likely a noteworthy plesiomorphy in *Acer*.

**Figure 9.**
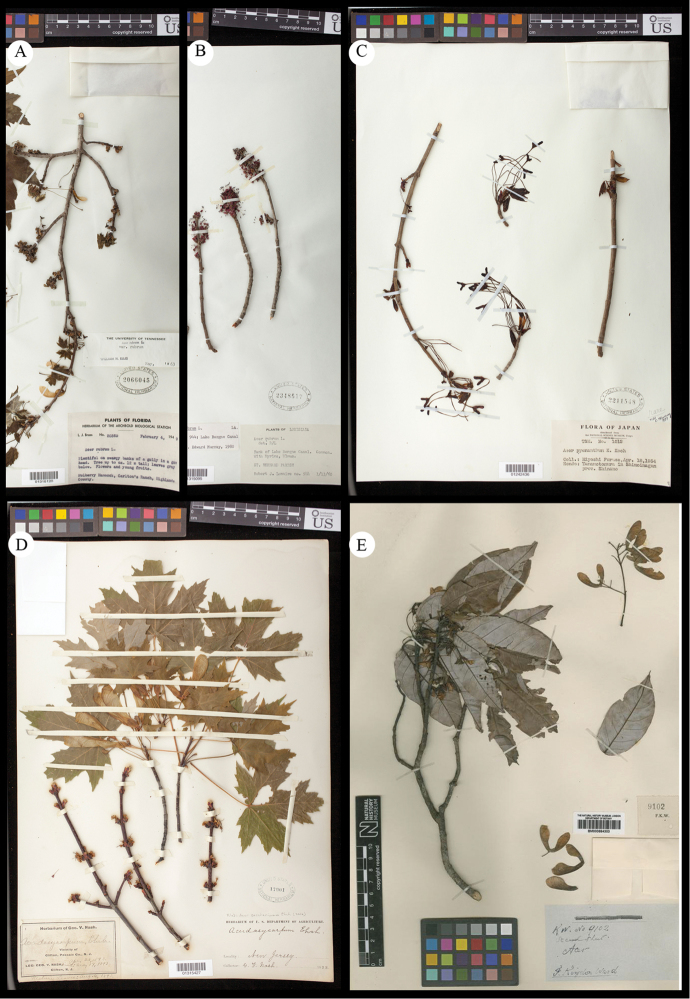
Inflorescences of *Acer* sects. *Rubra* and *Hyptiocarpa*. **A** *Acer rubrum* with umbels of pistilate flowers **B** *Acer rubrum* with umbels of staminate flowers **C** *A. pycnanthum* with umbels of pistilate flowers **C** *A. saccharinum* with umbels of pistilate flowers. Note flowers with two, divided persistent styles **D** *A. pinnatinervium* with racemose thyrse. Specimens in A–D deposited at US, and specimen in D deposited at the British National Museum (BM). Accession information visible in images, and detailed specimen records are available via the US online catalog (http://collections.nmnh.si.edu/search/botany/) and at the data portal of BM (http://data.nhm.ac.uk/).

Section Rubra s.l. has variable inflorescence architecture (Fig. 9). *Acer rubrum* (Fig. 9A–B), *A. pycnanthum* (Fig. 9C), and *A. saccharinum* (Fig. 9D) have inflorescences that are umbels (de Jong 1976; van Gelderen et al. 1994), while *A. laurinum* and allied taxa have inflorescences that may be racemes (*F.W. Junghuhn s.n.*, L) or paniculate thyrses (*Lindley, 418*, K) (de Jong 1976; van Gelderen et al. 1994). The umbels, which are unique within *Acer*, probably represent evolutionarily reduced racemes, while the racemes, which are more common in *Acer*, may represent reduced paniculate thyrses (de Jong 1976, Singer 2008). Inflorescences throughout sect. Rubra s.l. are almost exclusively lateral (Ohwi 1965, de Jong 1976, van Gelderen et al. 1994), although some authors report occasional terminal inflorescences in *A. pycnanthum* (van Gelderen et al. 1994; but contrast with *A. pycnanthum* in Ohwi 1965, de Jong 1976). While lateral inflorescences are common to other sections of *Acer*, exclusively lateral ones (or nearly so) occur only in sects. *Rubra*, *Lithocarpa*, and *Glabra*.

Species of sect. Rubra s.l. except *A. pycnanthum* may be monoecious or dioecious and exhibit labile sex expression among individuals (de Jong 1976; Primack and McCall 1986; Santamour 1993), and within-individual and within-clade labile sex expression occurs in some other groups of *Acer* and other Sapindaceae (Acevedo-Rodríguez et al. 2011; Renner et al. 2007). *Acer pycnanthum* is thought to be exclusively dioecious (de Jong 1976, Saeki 2008). Among monecious individuals of *Acer rubrum*, *A. saccharinum*, and *A. laurinum*, individual inflorescences are usually exclusively comprised of staminate or pistillate flowers. One prior study inferred that dioecy was ancestral in sect. Rubra s.l., but that inference was based on scoring *A. laurinum* as dioecious (Renner et al. 2007), which is not accurate (Bloembergen 1948; de Jong 1976; Xu et al. 2008). All flowers in sect. Rubra s.l. emerge from leafless buds, and this is a taxonomically informative trait that delimits some sections of *Acer* from others (de Jong 1976; van Gelderen et al. 1994).

**Figure 10.**
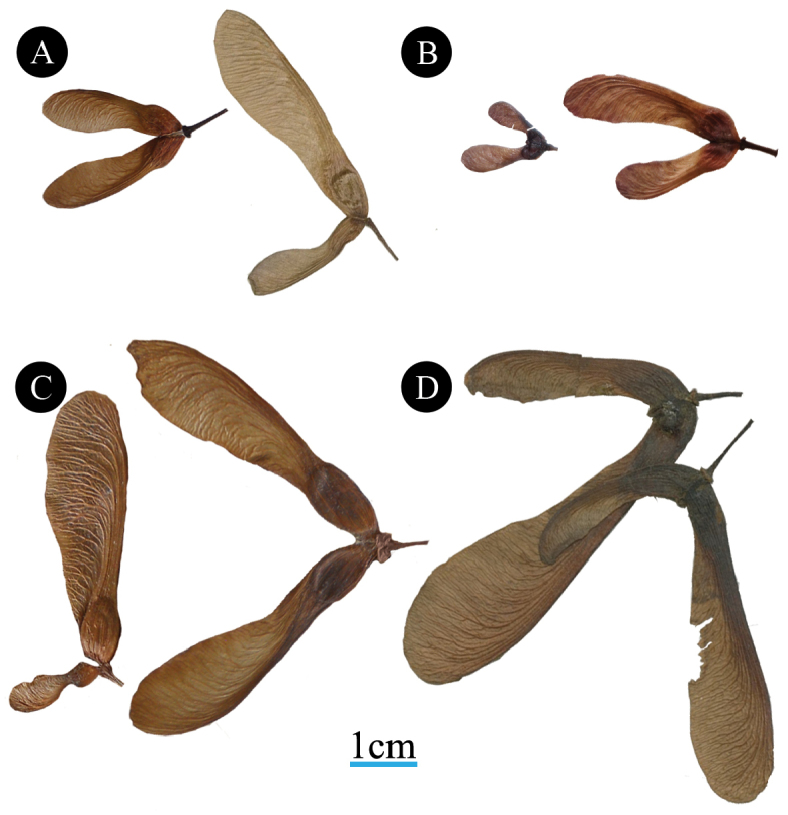
Fruits of species of Acer section Rubra. **A** *A. rubrum*. Specimen on left deposited at US National herbarium (US) with collection name and number: *Lilian 62*. Specimen on right deposited at Kew (K) as *Acer drummondii* Nutt. (= *A. rubrum*) with collection name and number: *Drummond 53*. Image of fruits obtained from image of specimen deposited in JSTOR Plants (http://plants.jstor.org/) **B** *A. pycnanthum*, used with attribution to Chinese Virtual Herbarium(http://www.cvh.ac.cn/); *Miyoshi Furuse 54050*, PE **C** *A. saccharinum* showing fruit with two fertilized ovules (upper) compared with one fertilized ovules and one partially developed, unfertilized ovule (lower). Specimens deposited at US with collection name and number: *Wolf s.n.* and *Pringle s.n.*, respectively **D** *A. laurinum* Specimen deposited at K with collection name and number: *Lindley, 418*. Image of fruits obtained from image of specimen deposited in JSTOR Plants. Scale bar of 1cm applies to all images.

Fruits in sect. Rubra s.l. also share many features (Fig. 10), especially from among those identified as taxonomically informative in a comprehensive study by Wolfe and Tanai (1987). We have observed that the fruits of all species in sect. Rubra have slightly inflated seed locules without keels, wings that are straight at the base, and mericarps forming an acute angle with respect to one another. Each of these traits tends to be monomorphic within sections. Each trait occurs in about half of all sections, but this suite of traits may be unique to sect. Rubra s.l. Additionally, species in sect. Rubra s.l. are capable of producing partially developed seedless mericarps (Fig. 10), compared to complete or extremely minimal (e.g., roughly pinhead-sized) development in other species and sections (de Jong 1976). The degree of development of seedless mericarps in *Acer* is well-characterized by de Jong (1976) and is taxonomically informative. Partially developed, seedless mericarps occur in about half of sections of *Acer*, and most sections are monomorphic for this trait. Fruits of sect. Rubra s.l. are highly variable in size within species with the largest fruits occurring in *A. saccharinum* and *A. laurinum* (Townsend 1972; van Gelderen et al. 1994; Xu et al. 2008).

Prior studies have proposed other plausible relationships for sect. Hyptiocarpa based on morphology. In particular, leaf morphology has often been used to link sect. Hyptiocarpa with *Acer oblongum* Wallich ex de Candolle and its close relatives in sect. Pentaphylla or *Integrifolia* (Pax 1885; Momotani 1962; Fang 1966). *Acer oblongum* has entire, unlobed elongate leaves and silvery abaxial surfaces (van Gelderen et al. 1994) that are similar to leaves in *A. laurinum*. Nevertheless, any association between *Acer oblongum* and sect. Hyptiocarpa has not been supported by molecular phylogenies, which show that *Acer oblongum* is associated with sect. Pentaphyllum and distant from sect. Rubra (Suh et al. 2000; Renner et al. 2008). Morphologically, *A. oblongum* differs from *A. laurinum* by having mostly terminal inflorescences and by flowers and leaves arising from the same buds (van Gelderen et al. 1994). Additionally, the waxes of *A. oblongum* may differ from those in sect. Rubra by extending partially onto the midrib. While we made this observation on many specimens at IBSC, we used a low magnification hand lens, and a more detailed study using higher magnification may be warranted. Another possible association for sect. Hyptiocarpa was with sect. Lithocarpa, which has a relatively large number of bud scales, axillary inflorescences from leafless buds, and insertion of stamens on a staminal disk (Ogata 1967); features that are also shared with sect. Rubra s.s., except for stamen insertion (Pax 1885; Ogata 1967; de Jong 1976; van Gelderen et al. 1994). In sect. Rubra stamens are inserted outside of the disk or the disk is absent in some individuals of each species (van Gelderen et al. 1994). Thus, the disk may be relatively labile within sect. Rubra s.s. and in sect. Rubra s.l. Section Hyptiocarpa differs from sect. Lithocarpa (except *A. macrophyllum* Pursh.) by having wood rays 3-4 cells wide rather than cells wide. Overall, in prior taxonomic work, recognition of a distinct sect. Hyptiocarpa, seems more motivated by uncertainties about its affinities (Ogata 1967; de Jong 1976; Delendick 1981; Wolfe and Tanai 1987; van Gelderen et al. 1994) than affirmation of its significant uniqueness within *Acer* (e.g., contrasted with *A. carpinifolium* and *A. negundo* Linnaeus).

### Section Rubra s.l. and evolutionary radiation

Section Rubra s.l. may have radiated out of the tropics and into temperate areas of Japan and North America based on our phylogenetic results (Fig. 2) and results presented in other molecular phylogenetic studies (Grimm et al. 2006; Renner et al. 2008). In particular, results suggest that *A. laurinum* is the earliest diverging species within sect. Rubra s.l. An out- of-the-tropics radiation in sect. Rubra s.l. may have been accompanied by, or even spurred by, polyploidization. According to the literature, *Acer laurinum* is diploid (2*n*=26), *A. saccharinum* is tetraploid, *A. pycnanthum* is hexaploid, and *A. rubrum* includes hexaploid, heptaploid (rarely), and octaploid individuals (Fig. 2), and these counts reflect attempts to avoid hybrid individuals and use materials originating from wild populations (Löve 1971; Santamour 1965; van Gelderen et al. 1994; Chromosome Count Database, http://ccdb.tau.ac.il/home/). Polyploidization is assumed to have played a role in adaptation to less equitable environments in some plant groups, and polyploidy sometimes shows clear positive correlation with latitude (Beaton and Hebert 1988). Future studies may examine the timing of evolutionary radiation, such as by using fossils and divergence time dating, to better understand possible correlations between ploidy level and past environments.

## Synopsis of Acer section Rubra s.l.

### Acer sect. Rubra

Taxon classificationPlantaeSapindalesSapindaceae

Pax, Bot. Jahrb. Syst. 6: 326. 1885.

Acer sect. Hyptiocarpa W.P. Fang, Acta Phytotax. Sin. 11: 172. 1966.
Acer sect. Laurina Ogata, Bull. Tokyo Univ. Forests 63: 151. 1967.#

#### Type species.

*Acer rubrum* Linnaeus.

#### Description.

Trees, deciduous or evergreen, with labile sex expression ranging from monoecy to dioecy (possibly exclusively dioecious in *A. pycnanthum*). Wood distinctly ring-porous, rays 1-4(10) cells wide. Bud scales imbricate, decussate, in pairs of 4-11. Leaves entire, unlobed, or 3- or 5- lobed, elliptic to ovate, toothed or entire, glaucous to blue-colored beneath; cuticular waxes of leaves comprising a smooth layer on the adaxial surface and bearing membranous platelets and wax splatter features abaxially; primary veins 1 or 3, 5 in 5-lobed individuals of *A. saccharinum*; petioles sometimes turning red (e.g., new growth, late season). Inflorescences axillary (rarely terminal) from leafless buds, usually emerging before leaves, paniculate thyrses, racemes, or umbels. Sepals 5. Petals 0 or 5, red, red-green, or green when present. Stamens 5-12, inserted on (*A. laurinum* and *A. pinnatinervium*) or outside of staminal disk, disk sometimes reduced or absent (*A. rubrum*, *A. saccharinum*, *A. pycnanthum*). Carpels 2. Fruits schizocarps with partially inflated seed locules, sometimes turning red during maturation, partitioning wall generally narrower than the seed locules; mericarps diverging from each other at less than 90°, wings straight to slightly convex on the proximal (vein-dense) side, curved on the distal side. Some fruits seedless and partially developed at maturity.

Five species showing a disjunct distribution between eastern and southeastern Asia (3 spp.) and eastern North America (2 spp.), a common biogeographic pattern among Northern Hemisphere plant groups (Donoghue and Smith 2004; Harris et al. 2013, 2017; Li 1952; Wen 1999, 2001; Xiang et al. 2015).

***Acer laurinum*** Hasskarl, Tijdschr. Natuurl. Gesch. Physiol. 10: 138. 1843.

*Acer javanicum* Junghuhn, 1841

*Acer niveum* Blume, 1847

*Acer cassiifolium* Blume, 1847 (as *cassiaefolium*)

*Acer philippinum* Merrill, 1906

*Acer garrettii* Craib, 1920

*Acer decandrum* Merrill, 1932

*Acer chionophyllum* Merrill, 1941

*Acer longicarpum* Hu & W. C. Cheng, 1948

*Acer macropterum* T. Z. Hsu & H. Sun, 1997

***Acer pinnatinervium*** Merrill, Brittonia 4: 109. 1941.

*Acer machilifolium* Hu & Cheng, 1948

*Acer jingdongense* T. Z. Hsu, 1983

***Acer pycnanthum*** K. Koch, Ann. Mus. Bot. Lugduno-Batavi 1: 250. 1864.

***Acer rubrum*** Linnaeus, Sp. Pl. 1055. 1753.

*Acer carolinianum* Walter, 1788

*Acer barbatum* Michaux, 1803, *pro parte*

*Acer sanguineum* Spach, 1834

*Saccharodendron barbatum* (Michaux) Nieuwland, 1914, *pro parte*

*Rufacer carolinianum* (Walter) Small, 1933

*Rufacer rubrum* (Linneaus) Small, 1933

***Acer saccharinum*** Linnaeus, Sp. Pl. 1055. 1753.

*Acer sylvestre* Young, 1783

*Acer glaucum* Marshall, 1785

Acer rubrum Linneaus var. pallidum Aiton

*Acer dasycarpum* Ehrhart, 1789

*Acer eriocarpum* Michaux, 1803

*Acer tomentosum* Steudel, 1821

*Acer coccineum* F. Michaux

*Saccharosphendamnus saccharina* (Linnaeus) Nieuwland, 1914

*Argentacer saccharinum* (Linnaeus) Small, 1933

## Conclusions

Based on evidence from molecular phylogeny, morphology, and leaf ultrastructure, we propose uniting sects. *Rubra* and *Hyptiocarpa* within Acer sect. Rubra. Our molecular phylogenetic results are in agreement with prior studies, which suggest that *Acer* sects. *Rubra* and *Hyptiocarpa* are sisters. Within these sections, species share taxonomically important characteristics including leaves with silvery abaxial surfaces resulting from similar cuticular wax structures, typically lateral inflorescences, labile sex expression, partial development of seedless fruits, and many aspects of fruit morphology. The unity of these sections yields better and more complete understanding their evolutionary and biogeographic history. We speculate that sect. Rubra s.l. radiated out of the tropics and that the radiation coincided with polyploidization.

## Supplementary Material

XML Treatment for Acer sect. Rubra

## References

[B1] Acevedo-Rodríguez P van Welzen PC Adema F van der Ham RWJM (2011) Sapindaceae. In: Kubitzki K (Ed.) Flowering Plants Eudicots: Sapindales, Cucurbitales, Myrtaceae. Springer, New York, 357–407.

[B2] Baker E (1974) The influence of environment on leaf wax development in Brassica oleracea var. gemmifera. New Phytologist 73: 955–966. https://doi.org/10.1111/j.1469-8137.1974.tb01324.x

[B3] Barrett R (2004) Maples. Firefly Books, Buffalo, New York, 96 pp.

[B4] Barthlott W Neinhuis C Cutler D Ditsch F Meusel I Theisen I Wilhelmi H (1998) Classification and terminology of plant epicuticular waxes. Botanical Journal of the Linnean Society 126: 237–260. https://doi.org/10.1111/j.1095-8339.1998.tb02529.x

[B5] Beaton MJ Hebert PDN (1988) Geographical parthenogenesis and polyploidy in *Daphnia pulex*. The American Naturalist 132: 837–845. https://doi.org/10.1086/284892

[B6] de Beaulieu ALH Mechelynck AL (2003) An Illustrated Guide to Maples. Timber Press, Portland, Oregon, 464 pp.

[B7] Bloembergen S (1948) Aceraceae. In: van Steenis CGGJ (Ed.) Flora Malesiana Series 1, Volume 4. Noordhoff-Kolff NV, Bjakarta, 3–4.

[B8] Blume CL (1847) Rumphia, Tomus Tertias. C. Rorat, Paris, 224 pp.

[B9] Boeger MRT Alves LC Negrelle RRB (2004) Leaf morphology of 89 tree species from a lowland tropical rain forest (Atlantic forest) in South Brazil. Brazilian Archives of Biology and Technology 47: 933–943. https://doi.org/10.1590/S1516-89132004000600013

[B10] Buerki S Lowry P Alvarez N Razafimandimbison SG Küpfer P Callmander MW (2010) Phylogeny and circumscription of Sapindaceae revisited: molecular sequence data, morphology and biogeography support recognition of a new family, Xanthoceraceae. Plant Ecology and Evolution 143: 148–159. https://doi.org/10.5091/plecevo.2010.437

[B11] Caddah MK Mayer JLS Bittrich V Amaral MDCED (2012) Species limits in the *Kielmeyera coriacea* complex (Calophyllaceae) – a multidisciplinary approach. Botanical Journal of the Linnean Society 168: 101–115. https://doi.org/10.1111/j.1095-8339.2011.01192.x

[B12] Clarke JM Richards RA (1988) The effects of glaucousness, epicuticular wax, leaf age, plant height, and growth environment on water loss rates of excised wheat leaves. Canadian Journal of Plant Science 68: 975–982. https://doi.org/10.4141/cjps88-118

[B13] Dehgan B (1980) Application of epidermal morphology to taxonomic delimitations in the genus *Jatropha* L. (Euphorbiaceae). Botanical Journal of the Linnean Society 80: 257–278. https://doi.org/10.1111/j.1095-8339.1980.tb01989.x

[B14] Delendick TJ (1981) A Systematic Review of the Aceraceae. Ph.D. Thesis, New York: City University of New York, 693 pp.

[B15] Dong W-P Liu J Yu J Wang L Zhou S-L (2012) Highly variable chloroplast markers for evaluating plant phylogeny at low taxonomic levels and for DNA barcoding. PLOS One 7: e35071. https://doi.org/10.1371/journal.pone.0035071 10.1371/journal.pone.0035071 PMC3325284 22511980

[B16] Donoghue MJ Smith SA (2004) Patterns in the assembly of the temperate forest around the Northern Hemisphere. Philosophical Transactions of the Royal Society of London: Biology 359: 1633–1644. https://doi.org/10.1098/rstb.2004.1538 10.1098/rstb.2004.1538 PMC1693435 15519978

[B17] Drummond A Rambaut A (2007) BEAST: Bayesian evolutionary analysis by sampling trees. BMC evolutionary biology 7: 214. https://doi.org/10.1186/1471-2148-7-214 10.1186/1471-2148-7-214 PMC2247476 17996036

[B18] Drummond AJ Suchard MA Xie D Rambaut A (2012) Bayesian phylogenetics with BEAUti and the BEAST 1.7. Molecular biology and evolution 29: 1969–1973. https://doi.org/10.1093/molbev/mss075 22367748 10.1093/molbev/mss075 PMC3408070

[B19] Edgar R (2004) MUSCLE: A multiple sequence alignment method with reduced time and space complexity. BMC Bioinformatics 5: 113. https://doi.org/10.1186/1471-2105-5-113 10.1186/1471-2105-5-113 PMC517706 15318951

[B20] Eglinton G Hamilton RJ (1967) Leaf epicuticular waxes. Science 156: 1322–1335. https://doi.org/10.1126/science.156.3780.1322 4975474 10.1126/science.156.3780.1322

[B21] Eigenbrode SD Espelie KE (1995) Effects of plant epicuticular lipids on insect herbivores. Annual Review of Entomology 40: 171–194. https://doi.org/10.1146/annurev.en.40.010195.001131

[B22] Fang W-P (1966) Revisio taxorum Aceracearum Sinicarum. Acta Phytotaxonomica Sinica 11: 139–189.

[B23] Federle W Maschwitz U Fiala B Riederer M Hölldobler B (1997) Slippery ant-plants and skillful climbers: selection and protection of specific ant partners by epicuticular wax blooms in *Macaranga* (Euphorbiaceae). Oecologia 112: 217–224. https://doi.org/10.1007/s004420050303 28307573 10.1007/s004420050303

[B24] Fernández V Guzmán-Delgado P Graça J Santos S Gil L (2016) Cuticle structure in relation to chemical composition: re-assessing the prevailing model. Frontiers in Plant Science 7: 427. https://doi.org/10.3389/fpls.2016.00427 10.3389/fpls.2016.00427 PMC4814898 27066059

[B25] van Gelderen DM de Jong PC Oterdoom HJ Dudley TR (1994) Maples of the World. Timber Press, Portland, Oregon, 458 pp.

[B26] Gorb E Voigt D Eigenbrode SD Gorb S (2008) Attachment force of the beetle *Cryptolaemus montrouzieri* (Coleoptera, Coccinellidae) on leaflet surfaces of mutants of the pea *Pisum sativum* (Fabaceae) with regular and reduced wax coverage. Arthropod-Plant Interactions 2: 247–259. https://doi.org/10.1007/s11829-008-9049-0

[B27] Grimm GW Renner SS Stamatakis A Hemleben V (2006) A nuclear ribosomal DNA phylogeny of *Acer* inferred with maximum likelihood, splits graphs, and motif analysis of 606 sequences. Evolutionary Bioinformatics 2: 7–22. PMC2674679 19455198

[B28] Haeckel E (1866) Der allgemeinen Entwickelungsgeschichte Generelle Phylogenie oder Allgemeine Entwickelungsgeschichte der organischen Stämme: Genealogie und Paläontologie. Zweiter Theil. Verlag von Georg Reimer, Berlin, 462 pp.

[B29] Harrington MG Edwards KJ Johnson SA Chase MW Gadek PA Manos PS (2005) Phylogenetic Inference in Sapindaceae *sensu lato* using plastid *matK* and *rbcL* DNA sequences. Systematic Botany 30: 366–382. https://doi.org/10.1600/0363644054223549

[B30] Harris AJ Chen P Xu X Yang X Wen J (2017) The phylogeny of Staphyleaceae reveals five major clades: Implications for generic delimitation and classical biogeographic disjunctions in the family. Journal of Systematics and Evolution. https://doi.org/10.1111/jse.12236

[B31] Harris AJ Frawley E Wen J (2017) The utility of single-copy nuclear genes for phylogenetic resolution of *Acer* and *Dipteronia* (Acereae, Sapindaceae). Annales Botanici Fennici 54: 209–222.

[B32] Harris AJ Wen J Xiang Q-Y (2013) Inferring the biogeographic origins of inter-continental disjunct endemics using a Bayes-DIVA approach. Journal of Systematics and Evolution 51: 117–133. https://doi.org/10.1111/jse.12007

[B33] Hasskarl C (1843) Annotations de plantis quibusdam Javanicis nonnullisque Japonicis, e catalogo horti Borgoriensis. Accedunt nonnullae novae species. Tijdschrift voor Natuurlijke Geschiedenis en Physiologie 10: 11–150.

[B34] Hasskarl C (1857) untitled. In: Junghuhn FR (Ed.) Java: Seine Gestalt, Pflanzendecke und innere Bauart Zweite Abtheilung. Arnoldishe Buchhandlung, Leipzig, 532.

[B35] Huelsenbeck JP Rannala B (2004) Frequentist properties of Bayesian posterior probabilities of phylogenetic trees under simple and complex substitution models. Systematic Biology 53: 904–913. https://doi.org/10.1080/10635150490522629 15764559 10.1080/10635150490522629

[B36] Jermiin LS Ho SY Ababneh F Robinson J Larkum AW (2004) The biasing effect of compositional heterogeneity on phylogenetic estimates may be underestimated. Systematic Biology 53: 638–643. https://doi.org/10.1080/10635150490468648 15371251 10.1080/10635150490468648

[B37] Junghuhn FR (1842) Fr. Junghuhn’s Abhandlungen. Monatsberichte über die Verhandlungen der Gesellschaft für Erdkunde zu Berlin 1841-42: 83–102.

[B38] de Jong PC (1976) Flowering and Sex Expression in *Acer* L.: A Biosystematic Study. Veenman & Zonen, Wageningen, The Netherlands, 201 pp.

[B39] Judd W Sanders R Donoghue M (1994) Angiosperm family pairs: preliminary phylogenetic analyses. Harvard Papers in Botany 5: 1–51.

[B40] Kearse M Moir R Wilson A Stones-Havas S Cheung M Sturrock S Buxton S Cooper A Markowitz S Duran C (2012) Geneious Basic: an integrated and extendable desktop software platform for the organization and analysis of sequence data. Bioinformatics 28: 1647–1649. https://doi.org/10.1093/bioinformatics/bts199 22543367 10.1093/bioinformatics/bts199 PMC3371832

[B41] Krause C (1978) Identification of four red maple cultivars with scanning electron microscopy. HortScience 13: 586–588.

[B42] Katoh K Misawa K Kuma K Miyata T (2002) MAFFT: a novel method for rapid multiple sequence alignment based on fast Fourier transform. Nucleic acids research 30: 3059–3066. https://doi.org/10.1093/nar/gkf436 12136088 10.1093/nar/gkf436 PMC135756

[B43] Katoh K Kuma K Toh H Miyata T (2005) MAFFT version 5: improvement in accuracy of multiple sequence alignment. Nucleic acids research 33: 511–518. https://doi.org/10.1093/nar/gki198 15661851 10.1093/nar/gki198 PMC548345

[B44] Larsson H Jaciw P (1967) Sap and syrup of five maple species. Research report 69. In: Ontario Department of Lands and Forests (Ed. ) Ottawa, Ontario, Canada, 62 pp.

[B45] Leaf Architecture Working Group (1999) The Manual of Leaf Architecture - morphological description and categorization of dicotyledonous and net-veined monocotyledonous angiosperms. Leaf Architecture Working Group, Washington, D.C., 67 pp.

[B46] Lee C Wen J (2004) Phylogeny of *Panax* using chloroplast *trnC*–*trnD* intergenic region and the utility of *trnC*–*trnD* in interspecific studies of plants. Molecular Phylogenetics and Evolution 31: 894–903. https://doi.org/10.1016/j.ympev.2003.10.009 15120387 10.1016/j.ympev.2003.10.009

[B47] Lee SB Suh MC (2015) Advances in the understanding of cuticular waxes in *Arabidopsis thaliana* and crop species. Plant Cell Reports 34: 557–572. https://doi.org/10.1007/s00299-015-1772-2 25693495 10.1007/s00299-015-1772-2

[B48] Li H-L (1952) Floristic relationships between eastern Asia and eastern North America. Transactions of the American Philosophical Society 42: 371–429. https://doi.org/10.2307/1005654

[B49] Li J-H Yue J-P Shoup S (2006) Phylogenetics of *Acer* (Aceroideae, Sapindaceae) based on nucleotide sequences of two chloroplast non-coding regions. Harvard Papers in Botany 11: 101–115. https://doi.org/10.3100/1043-4534(2006)11[101:POAASB]2.0.CO;2

[B50] Li D-Z Gao L-M Li H-T Wang H Ge X-J Liu J-Q Chen Z-D Zhou S-L Chen S-L (2011) Comparative analysis of a large dataset indicates that internal transcribed spacer (ITS) should be incorporated into the core barcode for seed plants. Proceedings of the National Academy of Sciences 108: 19641–19646. https://doi.org/10.1073/pnas.1104551108 10.1073/pnas.1104551108 PMC3241788 22100737

[B51] Löve Á (1971) IOPB Chromosome Number Reports XXXII. Taxon 20: 349–356.

[B52] Martin JT Juniper BE (1970) The Cuticles of Plants. St. Martin’s Press, New York, 347 pp.

[B53] Mayeux HS Jordan WR Meyer RE Meola SM (1981) Epicuticular wax on goldenweed (*Isocoma* spp.) leaves: variation with species and season. Weed Science 29: 389–393.

[B54] Merrill ED (1941) The upper Burma plants collected by Captain F. Kingdon Ward on the Vernay-Cutting Expedition. Brittonia 4: 20–188. https://doi.org/10.2307/2804985

[B55] Momotani (1962) Taxonomic study of the genus *Acer* with special reference to the seed proteins. III. System of *Acer*. Kyoto University Memoirs of the College of Science Series B 29: 177–189.

[B56] Mishler B (1988) Relations between ontogeny and phylogney with reference to bryophytes. In: Humphries CJ (Ed.) Ontogeny and Systematics. Columbia University Press, New York, 117–136.

[B57] Müller C (2008) Plant–Insect interactions on cuticular surfaces. In: Reiderer M, Müller C (Eds) Annual Plant Reviews, Biology of the Plant Cuticle, 398–422.

[B58] Murray AE (1970) A Monograph of the Aceraceae. Ph.D. Thesis, State College, PA: Pennsylvania State University, 337 pp

[B59] Ogata K (1967) A systematic study of the genus *Acer*. Bulletin of the Tokyo University of Forests 63: 89–206.

[B60] Ohwi J (1965) Flora of Japan (in English). Smithsonian Institution, Washington, D.C., 1067 pp.

[B61] Pan Y-Z Gong X Yang Y (2008) Phylogenetic position of the genus *Dobinea*: Evidence from nucleotide sequences of the chloroplast gene *rbcL* and the nuclear ribosomal ITS region. Journal of Systematics and Evolution 49: 586–594.

[B62] Swofford DL (2002) PAUP*: Phylogenetic Analysis Using Parsimony. v.v. 4.0 b10

[B63] Pathan AK Bond J Gaskin RE (2010) Sample preparation for SEM of plant surfaces. Materials Today 12: 32–43. https://doi.org/10.1016/S1369-7021(10)70143-7

[B64] Pax FA (1885) Monographie der Gattung Acer. Wilhelm Engelmann, Leipzig, 87 pp.

[B65] Penn O Privman E Ashkenazy H Landan G Graur D Pupko T (2010) GUIDANCE: a web server for assessing alignment confidence scores. Nucleic acids research 38: W23–W28. https://doi.org/10.1093/nar/gkq443 10.1093/nar/gkq443 PMC2896199 20497997

[B66] Primack RB McCall C (1986) Gender variation in a red maple population (*Acer rubrum*; Aceraceae): a seven-year study of a “polygamodioecious” species. American Journal of Botany 73: 1239–1248. https://doi.org/10.2307/2444057

[B67] Prusinkiewicz P Erasmus Y Lane B Harder LD Coen E (2007) Evolution and development of inflorescence architectures. Science 316: 1452–1456. https://doi.org/10.1126/science.1140429 17525303 10.1126/science.1140429

[B68] Punnuri S Harris-Shultz K Knoll J Ni X Wang H (2017) The genes *bm2* and *blmc* that affect epicuticular wax deposition in *Sorghum* are allelic. Crop Science 57: 1552–1556. https://doi.org/10.2135/cropsci2016.11.0937

[B69] Qian H Ricklefs RE (2000) Large-scale processes and the Asian bias in species diversity of temperate plants. Nature 407: 180–182. https://doi.org/10.1038/35025052 11001054 10.1038/35025052

[B70] Rambaut A Drummond A (2007) Tracer. 1.4 ed. http://beast.bio.ed.ac.uk/Tracer

[B71] Rambaut A Drummond A (2009) FigTree v1. 4.0: Tree figure drawing tool. Raven PH (1972) Plant species disjunctions: a summary. Annals of the Missouri Botanical Garden 59: 234–246.

[B72] Renner S Beenken L Grimm G Kocyan A Ricklefs R (2007) The evolution of dioecy, heterodichogamy, and labile sex expression in *Acer*. Evolution 61: 2701–2719. https://doi.org/10.1111/j.1558-5646.2007.00221.x 17894810 10.1111/j.1558-5646.2007.00221.x

[B73] Renner SS Grimm GW Schneeweiss GM Stuessy TF Ricklefs RE (2008) Rooting and dating maples (*Acer*) with an uncorrelated-rates molecular clock: implications for North American/Asian disjunctions. Systematic Biology 57: 795–808. https://doi.org/10.1080/10635150802422282 18853365 10.1080/10635150802422282

[B74] Ronquist F Huelsenbeck J Teslenko M (2011) MrBayes version 3.2 Manual: Tutorials and Model Summaries. http://mrbayes.sourceforge.net/mb3.2_manual.pdf

[B75] Ronquist F Huelsenbeck JP (2003) MrBayes 3: Bayesian phylogenetic inference under mixed models. Bioinformatics 19: 1572–1574. https://doi.org/10.1093/bioinformatics/btg180 12912839 10.1093/bioinformatics/btg180

[B76] Ronquist F Teslenko M van der Mark P Ayres DL Darling A Höhna S Larget B Liu L Suchard MA Huelsenbeck JP (2012) MrBayes 3.2: Efficient Bayesian phylogenetic inference and model choice across a large model space. Systematic Biology 61: 539–542. https://doi.org/10.1093/sysbio/sys029 22357727 10.1093/sysbio/sys029 PMC3329765

[B77] Saeki I (2008) Sexual reproductive biology of the endangered Japanese red maple (*Acer pycnanthum*). Ecological Research 23: 719–727.https://doi.org/10.1007/s11284-007-0431-7

[B78] Sela I Ashkenazy H Katoh K Pupko T (2015) GUIDANCE2: accurate detection of unreliable alignment regions accounting for the uncertainty of multiple parameters. Nucleic Acids Research 43: W7–W14. https://doi.org/10.1093/nar/gkv318 10.1093/nar/gkv318 PMC4489236 25883146

[B79] Santamour F (1993) Freeman maple-illusion and truth. Journal of Arboriculture 19: 195–195.

[B80] Santamour FS (1965) Cytological studies in red and silver maples and their hybrids. Bulletin of the Torrey Botanical Club, 127–134. https://doi.org/10.2307/2483933

[B81] Sargent CS (1922) Manual of the Trees of North America (Exclusive of Mexico). Houghton Mifflin, New York, 910 pp https://doi.org/10.5962/bhl.title.50215

[B82] Shaw J Lickey EB Beck JT Farmer SB Liu W Miller J Siripun KC Winder CT Schilling E Small RL (2005) The tortoise and the hare II: relative utility of 21 noncoding chloroplast DNA sequences for phylogenetic analysis. American Journal of Botany 92: 142–166. https://doi.org/10.3732/ajb.92.1.142 21652394 10.3732/ajb.92.1.142

[B83] Sheffield NC (2013) The interaction between base compositional heterogeneity and among-site rate variation in models of molecular evolution. ISRN Evolutionary Biology 2013: 1–8. https://doi.org/10.5402/2013/391561

[B84] Singer SR (2008) Inflorescence architecture—moving beyond description to development, genes and evolution. In: Ainsworth C (Ed.) Flowering and its Manipulation, Annual Plant Reviews Volume 20. Blackwell Publishing, Ames, Iowa, 98–111.

[B85] Suh Y Heo K Park C-W (2000) Phylogenetic relationships of maples (*Acer* L.; Aceraceae) implied by nuclear ribosomal ITS sequences. Journal of Plant Research 113: 193–202. https://doi.org/10.1007/PL00013914

[B86] Sutter E Langhans RW (1982) Formation of epicuticular wax and its effect on water loss in cabbage plants regenerated from shoot-tip culture. Canadian Journal of Botany 60: 2896–2902. https://doi.org/10.1139/b82-350

[B87] Tamura K Stecher G Peterson D Filipski A Kumar S (2013) MEGA6: molecular evolutionary genetics analysis version 6.0. Molecular biology and evolution 30: 2725–2729. https://doi.org/10.1093/molbev/mst197 24132122 10.1093/molbev/mst197 PMC3840312

[B88] Tanai T (1978) Taxonomical investigation of the living species of the genus *Acer* L., based on vein architecture of leaves. Journal of the Faculty of Science, Hokkaido University Series 4, Geology and Mineralogy 18: 243–282.

[B89] Tarrío R Rodríguez-Trelles F Ayala FJ (2000) Tree rooting with outgroups when they differ in their nucleotide composition from the ingroup: the *Drosophila saltans* and *willistoni* groups, a case study. Molecular Phylogenetics and Evolution 16: 344–349. https://doi.org/10.1006/mpev.2000.0813 10991788 10.1006/mpev.2000.0813

[B90] Thompson JD Higgins DG Gibson TJ (1994) CLUSTAL W: improving the sensitivity of progressive multiple sequence alignment through sequence weighting, position-specific gap penalties and weight matrix choice. Nucleic Acids Res 22: 4673–4680.https://doi.org/10.1093/nar/22.22.4673 7984417 10.1093/nar/22.22.4673 PMC308517

[B91] Thorne R (1972) Major disjunctions in the geographic ranges of seed plants. The Quarterly Review of Biology 47: 365–411. https://doi.org/10.1086/407399

[B92] Tian X Guo Z-H Li D-Z (2002) Phylogeny of Aceraceae based on ITS and *trnL-F* data sets. Acta Botanica Sinica 44: 714–724.

[B93] Townsend AM (1972) Geographic variation in fruit characteristics of *Acer rubrum* Bulletin of the Torrey Botanical Club, 122–126. https://doi.org/10.2307/2484691

[B94] Vaidya G Lohman DJ Meier R (2011) SequenceMatrix: concatenation software for the fast assembly of multi-gene datasets with character set and codon information. Cladistics 27: 171–180. https://doi.org/10.1111/j.1096-0031.2010.00329.x 10.1111/j.1096-0031.2010.00329.x 34875773

[B95] Waterhouse AM Procter JB Martin DMA Clamp M Barton GJ (2009) Jalview Version 2—a multiple sequence alignment editor and analysis workbench. Bioinformatics 25: 1189–1191. https://doi.org/10.1093/bioinformatics/btp033 19151095 10.1093/bioinformatics/btp033 PMC2672624

[B96] Weakley AS (2011) Flora of the Southern and Mid-Atlantic States. University of North Caro lina Herbarium, North Carolina Botanical Garden, University of North Carolina, Chapel Hill, 1320 pp.

[B97] Wen J (1999) Evolution of the eastern Asian and eastern North American disjunct distributions in flowering plants. Annual Review of Ecology and Systematics 30: 421–455. https://doi.org/10.1146/annurev.ecolsys.30.1.421

[B98] Wen J (2001) Evolution of Eastern Asian–Eastern North American biogeographic disjunctions: A few additional issues. International Journal of Plant Sciences 162: S117–S122. https://doi.org/10.1086/322940

[B99] Wen J (2011) Systematics and biogeography of *Aralia* L. (Araliaceae): Revision of *Aralia* sects. *Aralia*, *Humiles*, *Nanae*, and *Sciadodendron*. Contributions from the United States National Herbarium, 57: 1–172.

[B100] Wen J Nie Z-L Ickert-Bond SM (2016) Intercontinental disjunctions between eastern Asia and western North America in vascular plants highlight the biogeographic importance of the Bering land bridge from late Cretaceous to Neogene. Journal of Systematics and Evolution 54: 469–490. https://doi.org/10.1111/jse.12222

[B101] Willis JC (1980) A dictionary of the flowering plants and ferns. 8th Edition. Cambridge University Press, Cambridge, 1214 pp.

[B102] Wissemann V (2000) Epicuticular wax morphology and the taxonomy of Rosa (section Caninae, subsection Rubiginosae). Plant Systematics and Evolution 221: 107–112. https://doi.org/10.1007/BF01086384

[B103] Wolfe JA Tanai T (1987) Systematics, phylogeny, and distribution of *Acer* (maples) in the Cenozoic of western North America. Journal of the Faculty of Science, Hokkaido University Series 4, Geology and Mineralogy 22: 1–246.

[B104] Xiang JY Wen J Peng H (2015) Evolution of the eastern Asian–North American biogeographic disjunctions in ferns and lycophytes. Journal of Systematics and Evolution, 53: 2–32. https://doi.org/10.1111/jse.12141

[B105] Xu T Chen Y-S de Jong PC Oterdoom HJ Chang C-S (2008) Aceraceae. In: Wu ZY Raven PH Hong DY (Eds) Flora of China Vol 11 (Oxalidaceae through Aceraceae). Missouri Botanical Garden Press, St. Louis, 515–553.

[B106] Yang Z (1996) Among-site rate variation and its impact on phylogenetic analyses. Trends in Ecology & Evolution 11: 367–372. https://doi.org/10.1016/0169-5347(96)10041-0 21237881 10.1016/0169-5347(96)10041-0

[B107] Zuo Y-J Chen Z-J Kondo K Funamoto T Wen J Zhou S-L (2011) DNA barcoding of *Panax* species. Planta Medica 77: 182–187. https://doi.org/10.1055/s-0030-1250166 20803416 10.1055/s-0030-1250166

[B108] Zuo Y-J Wen J Zhou S-L (2017) Intercontinental and intracontinental biogeography of the eastern Asian – eastern North American disjunct *Panax* (the ginseng genus, Araliaceae), emphasizing its diversification processes in eastern Asia. Molecular Phylogenetics and Evolution. https://doi.org/10.1016/j.ympev.2017.06.016 10.1016/j.ympev.2017.06.016 28743642

